# Codesigning a pharmacist-led transition of care program in the emergency department: a qualitative participatory study

**DOI:** 10.1007/s11096-026-02210-3

**Published:** 2026-08-14

**Authors:** Eman Alhmoud, Raja Barazi, Binny Thomas, Almunzer Zakaria, Lubna Andraous, Rasha El Enany, Palli Valapila Abdulrouf, Sameer Pathan, Hamde Nazar, Mohamed Izham Mohamed Ibrahim, Muhammad Abdul Hadi

**Affiliations:** 1 QU Health Sector, Qatar University, Doha, Qatar; 2 Hamad Medical Corporation, Doha, Qatar; 3 National Institute for Health Research (NIHR) Newcastle Patient Safety Research Collaboration, Newcastle University, Newcastle-Upon-Tyne, UK; 4 Department of Clinical Pharmacy and Practice, College of Pharmacy, QU Health, Qatar University, P.O. Box 2713, Doha, Qatar; 5 School of Pharmacy and Biomolecular Sciences, Royal College of Surgeons Ireland, Dublin, Ireland

**Keywords:** Co-design, Community-based participatory research, Emergency Service, Hospital, Pharmacy Service, Hospital, Qualitative research, Transitional care

## Abstract

**Introduction:**

Transitions from the emergency department (ED) to home are high-risk periods for medication-related harm. Pharmacist-led interventions may improve medication safety and care continuity, however, co-designed approaches to developing such interventions remain limited.

**Aim:**

This study aimed to co-design a pharmacist-led transition-of-care (ToC) program to improve medication management for adults discharged from the ED, with the intention of subsequently evaluating it in a pilot randomized controlled trial.

**Method:**

A two-phase qualitative study was undertaken at a general teaching hospital in Qatar within a Participatory Health Research (PHR) framework. Phase I involved one-to-one interviews and focus groups with patients, pharmacists, physicians, and nurses. Phase II consisted of a co-design workshop with decision makers, including departmental leaders and hospital administrators, followed by structured refinement through additional stakeholder meetings and an electronic prioritization survey. Data were analyzed using an inductive-deductive approach, guided by two theoretical frameworks, the Theoretical Domains Framework and the Care Transitions Framework. Five proposed interventions were evaluated against the APEASE (Acceptability, Practicability, Effectiveness, Affordability, Safety, Equity) criteria, and identified barriers were mapped to corresponding intervention functions and implementation strategies.

**Results:**

Phase I, comprising three focus groups with physicians (n = 6), nurses (n = 7), and clinical pharmacists (n = 6), and four interviews with two patients and two Patient and Family Advisory Council (PFAC) representatives, identified barriers, enablers, and stakeholder priorities related to medicines management that informed the Phase II co-design workshop. Stakeholders rated the five proposed interventions: discharge medication reconciliation, prescription review, patient counseling and education, pharmacist-to-pharmacist handover, and post-discharge follow-up with medication review, as highly feasible (mean 83%). All intervention components were retained due to their interdependence. Eligibility criteria for this intervention were decided as: patients discharged with new chronic diagnoses, newly prescribed high-risk medications, polypharmacy (i.e., taking ≥ 5 regular medications), and chronic disease exacerbations. A standardized workflow was co-developed, outlining patient identification and communication. Stakeholders prioritized key intervention functions and implementation strategies via structured voting.

**Conclusion:**

The pharmacist-led transition-of-care program blueprint was co-designed using a participatory, theory-informed process. The finalized program will undergo feasibility testing through a pilot randomized controlled trial and a process evaluation.

**Supplementary Information:**

The online version contains supplementary material available at https://doi.org/10.1007/s11096-026-02210-3.

## Impact statements

- Participatory development ensures the resulting programme reflects genuine patient needs and discharge experiences.
- Targeted eligibility criteria can help pharmacy services prioritize patients most likely to benefit from transition-of-care interventions, improving the feasibility of service delivery.
- Co-designed implementation tools are likely to reduce the evidence-to-practice gap for emergency department transition of care services.

## Introduction

Medication-related harm, defined as “the harm caused by medication if taken incorrectly, monitored insufficiently, or as the result of an error, accident, or communication problem” [1], represents a major patient safety challenge, particularly during care transitions from hospital to community settings [2, 3]. Evidence suggests that up to one in two adults experience at least one medication error or unintentional medication discrepancy following hospital discharge, and one in five suffers an adverse drug event (ADE) [3].

Discharge from the emergency department (ED) to the community presents unique challenges, with an estimated 40% of ED-discharged patients experiencing at least one medication-related problem, not identified during the ED stay. These include prescribing errors (46.8%) and issues related to patient adherence or knowledge (31.2%), with 49.4% rated as moderate-to-high significance, and often involving high-risk medications such as strong opioids, insulin, and anticoagulants [4]. Furthermore, challenges in securing timely follow-up with primary care providers compound these risks [5–7]. Up to one-fifth of patients discharged with exacerbations of asthma, Chronic Obstructive Pulmonary Disease (COPD), or Congestive Heart Failure CHF) return to the ED within 30-days [8]. Pharmacists can play a critical role in improving medication safety during care transitions, as evidenced in the literature [9–16]. Compared to usual care, pharmacist-led transitional care (ToC) interventions targeting patients discharged from the hospital have consistently been shown to reduce medication discrepancies, medication errors, and adverse drug events (ADEs) [12–15]. Furthermore, these interventions have been associated with clinically meaningful reductions in 30-day all-cause hospital readmissions [10, 16].

Despite the substantial body of literature evaluating pharmacist-led transitional care interventions at hospital discharge, the evidence specific to pharmacist-delivered interventions in the ED remains limited and emergent [17–19]. Existing studies have been criticized for several methodological and reporting limitations, including small sample sizes, absence of control groups, non-randomized study designs, and missing appropriate outcome data due to high attrition rates [8, 20, 21]. In particular, the lack of transparent reporting on how these interventions were developed and implemented undermines their reproducibility and reduces their potential for real world uptake [22]. Furthermore, the limited engagement of key stakeholders (e.g., patients and healthcare professionals) during the intervention development process [23, 24] inherently risks that these interventions will fail to address user needs and preferences, thereby compromising their relevance and acceptability.

There is growing recognition of utilizing co-design approaches [25–27] in the development and implementation of healthcare interventions with an increasing application in pharmacy practice research [28–31]. The complexity and distinctive operational challenges of ED settings, which often limit the adoption of change [32], highlight the importance of genuine stakeholder involvement in the decision-making, and a comprehensive understanding of contextual factors influencing implementation. Co-designing an intervention ensures that interventions are grounded in lived experiences and professional insights, leading to improved user experience, efficiency, and clinical outcomes [33].

### Aim

The current study aimed to co-design a pharmacist-led ToC program for adults discharged from the ED, with the intention of subsequently evaluating it in a pilot randomized controlled trial (RCT).

## Method

### Design

A two-phase qualitative study was undertaken within a Participatory Health Research (PHR) framework [34]. Phase I aimed to explore the experiences and contextual factors influencing medication management during the transition from the ED to the community, and to identify the barriers, enablers, and priorities that would shape intervention development in Phase II. Phase II aimed to translate these findings into a feasible, acceptable, and contextually appropriate pharmacist-led ToC program through collaborative co-design with patients, healthcare professionals, and decision-makers. Co-design principles of inclusivity, participation, equity, iterative refinement, and transparency guided all phases of the study [35].

The intervention development process was reported in accordance with the guidance for reporting intervention development studies in health research (GUIDED) [36] (Supplementary file 1, Table S1), with qualitative reporting informed by the Consolidated Criteria for Reporting Qualitative Research (COREQ) checklist [37] (Supplementary file 1, Table S2).

The study design and methods have been summarized below; a detailed protocol is presented elsewhere [38].

### Study setting

The study was conducted at an 85-bed ED within a general teaching hospital under Hamad Medical Corporation (HMC), Qatar's main public healthcare provider. The ED serves approximately 161,000 patients annually and is supported by a single clinical pharmacist, primarily covering critical care areas. At the time of the study, patients discharged from the ED were not routinely referred to the hospital-based pharmacist-led medication therapy management (MTM) clinic, and no formal handover process existed between ED and MTM pharmacists. The MTM clinic is provided free of charge to all patients. In Qatar, pharmacists do not have independent prescribing authority but may initiate selected medication or laboratory orders under collaborative practice agreements with physician co-signature. The hospital uses the Cerner® electronic health record system, integrated across HMC and the Primary Health Care Corporation (PHCC) facilities.

### Participants

The participant recruitment was guided by the stakeholder mapping using the influence-benefit grid [39] (Supplementary file 1, Table S3), targeting individuals with high influence and benefit in relation to the ED ToC. Participants were identified through purposive and snowball sampling.

Phase I engaged two participant groups: (i) adult patients or patient representatives recently discharged from the ED, including members of the hospital's Patient and Family Advisory Council (PFAC), with prior ED discharge experience; and (ii) healthcare professionals directly involved in ED ToC, including clinical pharmacists, physicians, and nurses, purposively selected to achieve diversity in age, gender, nationality, and years of experience. Phase II comprised selected representatives from phase I alongside key decision makers, including departmental leaders and hospital administrators. Only Arabic- or English-speaking participants were eligible.

Eligible ED patients were identified by the research team through real-time monitoring and retrospective review of electronic records and were approached either during their ED visit or by telephone within one week of discharge. PFAC members were identified through the PFAC membership list and contacted by phone. Healthcare professionals were identified through professional networks and departmental lists and invited by email. All potential participants received a Participant Information Sheet outlining the study details prior to enrolment.

Sample size was guided by the concept of information power rather than traditional data saturation, given the focused study aim, purposive recruitment of information-rich stakeholder groups, and theory-informed analysis [24, 25].

### Theoretical underpinning

Multiple theoretical frameworks were purposively applied, each serving a distinct, predefined function at a specific stage of intervention development. Theoretical Domains Framework (TDF) [40] and Care Transitions Framework (CTF) [41] informed data collection and analysis, capturing behavioural and organisational determinants of medication management at ED-to-home transitions, respectively.

The APEASE criteria (Affordability, Practicability, Effectiveness, Acceptability, Safety, and Equity) [42, 43] provided a structured framework for stakeholder appraisal of proposed intervention components.

Identified barriers were subsequently mapped to theory- and evidence-informed implementation strategies using the SELECT tool [44], which draws on the COM-B (Capability, Opportunity, Motivation–Behaviour) model and TDF to match identified barriers to theoretically linked implementation strategies, and the CFIR-ERIC Implementation Strategy Matching Tool [45], which links barriers identified using the Consolidated Framework for Implementation Research (CFIR), the parent framework of the CTF used in this study, to discrete, evidence-based implementation strategies from the Expert Recommendations for Implementing Change (ERIC) compilation.

### Data collection

#### **Phase I**

Data were collected between February and March 2025 through uni-professional focus groups with healthcare professionals and one-to-one interviews with patients or their representatives. One-to-one interviews offered a private setting, while uni-professional focus groups facilitated open discussion and minimized interprofessional power imbalances.

A stakeholder-specific topic guide, informed by the TDF [40] and CTF [41], structured all enquiries; guides were published with the study protocol [38] and are available in the online supplementary material (Supplementary file 2).

Ground rules were announced at the beginning of each focus group to promote mutual respect, encourage open sharing of perspectives, and ensure equal contribution from all participants. Focus groups were facilitated by EH (a female pharmacy leader at the study site) as moderator, with RB (a female clinical pharmacist at the study site) or MAH (a male Professor of Clinical Pharmacy and Practice and research mentor) serving as note takers. Interviews were conducted by EH, who was acquainted with the clinical pharmacists but not with other stakeholders. All research team members had qualitative research experience.

Discussions explored medication management challenges around ED discharge, potential program users, and the feasibility of five proposed intervention components, derived from a prior systematic review [46] and professional guidelines [47, 48]. Feasibility was evaluated using the APEASE criteria [42, 43] as a structured discussion framework. The five intervention components included: (1) medication history taking and discharge medication reconciliation; (2) review of discharge prescription; (3) patient education and self-management support at discharge; (4) discharge planning, structured hand-offs and coordination of post-discharge appointments; and (5) pharmacist-led post-discharge follow-up in the MTM clinic.

Healthcare professionals rated each APEASE criterion on a five-point scale (1 = lowest, 5 = highest) and discussed their ratings until unanimous agreement was reached.

Given that the handoff component involved communication between ED and ambulatory care clinical pharmacists providing the pharmacist-led MTM service, APEASE ratings for this component were completed exclusively by pharmacists from both services. Patient representatives provided narrative feedback on proposed components. Facilitated discussions further explored implementation barriers, enablers, anticipated unintended consequences, and practical suggestions for integrating interventions into routine workflows.

#### **Phase II**

Comprised a two-hour in-person co-design workshop held at the study site in May 2025, involving nine participants. Prior to the session, participants received a briefing pack summarizing key Phase I findings, proposed intervention components, and workshop objectives. The workshop was structured into four sequential activities. First, the Phase I findings, including identified barriers, enablers, and stakeholder priorities, were presented to ground participants in the contextual evidence base. Second, participants engaged in facilitated group discussions to define eligibility criteria for the intervention. Third, intervention components were prioritized based on feasibility ratings conducted in Phase I and identified contextual needs. Fourth, intervention component-specific workflows were developed collaboratively. The session included consensus-building activities to agree on a preliminary prototype of the pharmacist-led ToC program; any unresolved disagreements were addressed through discussion until unanimous agreement was reached. The workshop was facilitated by EH, with MAH serving as a note-taker.

Discussions were captured through audio recording and detailed field notes, supplemented by participant-generated outputs including ranking sheets.

Following the workshop, a list of implementation strategies derived from Phase I (described later) was circulated to participants via an electronic survey (Microsoft Forms) for structured voting and prioritization, as time constraints precluded completing this exercise during the workshop.

#### **Post-Phase II follow-up**

The program workflow and implementation resources were iteratively developed and refined through stakeholder co-design activities and targeted operational discussions, including four meetings with clinical pharmacists, one meeting with ED leads, three interviews with hospital leaders, and email communications. Outputs were subsequently synthesized into a prototype intervention and a logic model, and disseminated to key stakeholders.

### Data analysis

All sessions were audio-recorded and transcribed verbatim. Phase I transcripts were independently analyzed by two experienced qualitative researchers (EH, RB) using: (1) inductive thematic analysis following Braun and Clarke six-step approach [49], and (2) deductive framework analysis, mapping participant responses to relevant TDF and CTF domains [50]. Coding and interpretive discrepancies were resolved through iterative team discussion and consensus. Analytical rigor was further enhanced through reflexive discussions, triangulation across stakeholder groups, and an audit trail documenting methodological and analytical decisions. Additionally, member checking was undertaken by sharing phase I summary findings with all 23 participants; no corrections or alternative interpretations were submitted in response.

Data from the co-design workshop and electronic prioritization survey were analyzed descriptively to document the workshop outputs. Implementation strategies achieving 100% stakeholder agreement in the survey were adopted.

Phase I findings informed two complementary outputs: (1) theory- and evidence-informed implementation strategies to support overall program adoption and integration into routine care, developed thorough implementation strategy mapping (described below); and (2) intervention-specific workflow specifications, refined through Phase II co-design and post-workshop operationalization (described under Data Collection).

### Implementation strategy mapping

Identified implementation barriers were mapped to theory- and evidence-informed intervention functions and implementation strategies at individual and organizational levels using the SELECT tool [44] and the CFIR-ERIC Implementation Strategy Matching Tool [45], as described above.

**Fig. 1:**
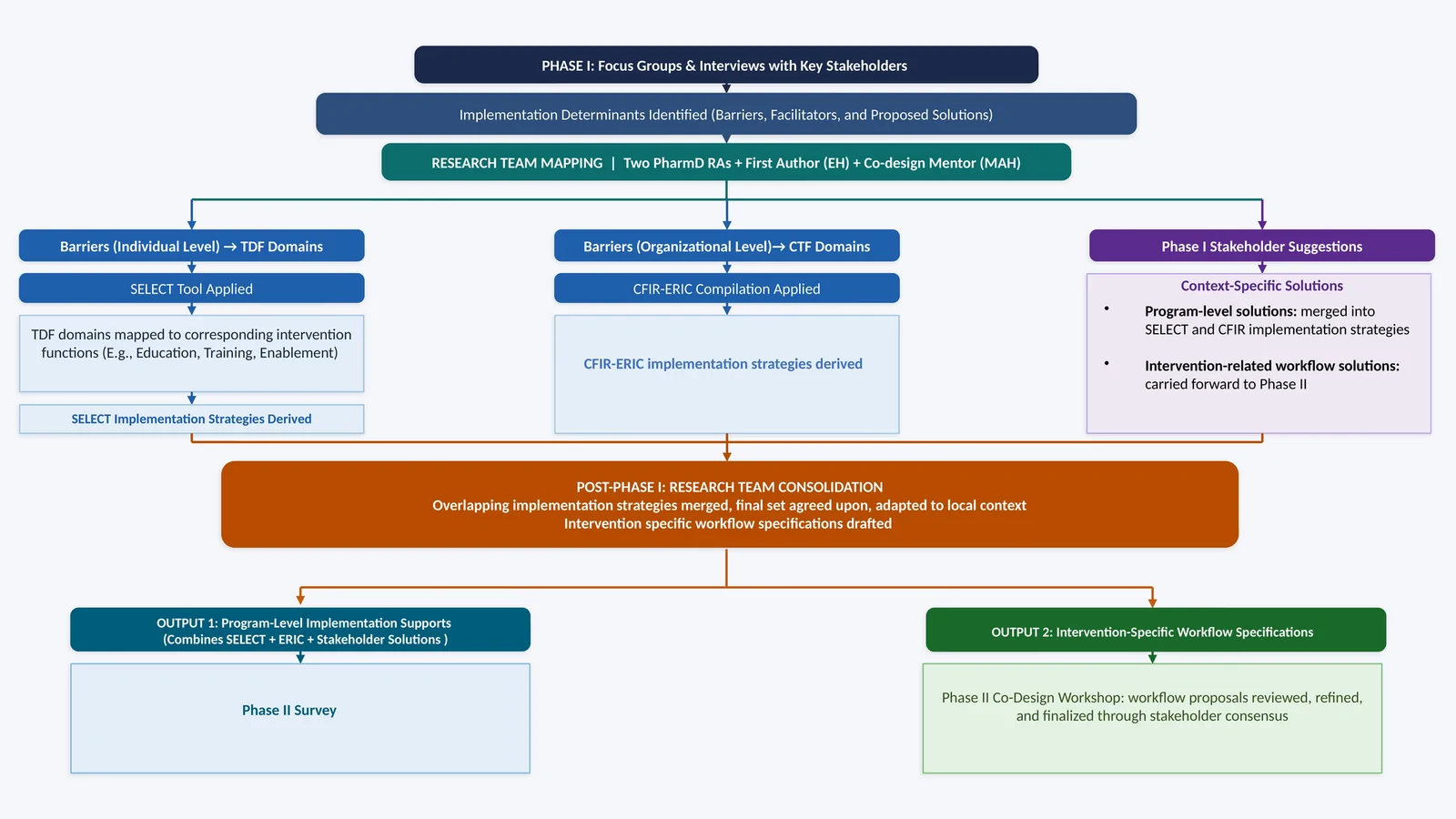
Analytical pathway from Phase I determinants (barriers and facilitators) to intervention specifications and implementation strategies

Stakeholder-proposed strategies and workflow solutions were incorporated throughout. Initial mapping was conducted independently by two trained research assistants (PharmD graduates) and subsequently refined through iterative discussions with the first author (EH), a clinical pharmacy leader at the study site, and a research mentor with co-design expertise (MAH). Overlapping strategies were consolidated, adapted to the local context, and synthesized into a final set of implementation strategies. Figure 1 illustrates the analytical pathway from Phase I stakeholder determinants to co-designed intervention specifications and implementation strategies.

### Ethics approval

This study was performed in line with the principles of the Declaration of Helsinki.

Approval was granted by the Medical Research Centre of Hamad Medical Corporation, Qatar (MRC-01-24-699) and Qatar University Institutional Review Board (QU-IRB 009/2025-EM).

## Results

### Participant characteristics and baseline demographics

#### **Phase I**

A total of 27 participants were invited; 23 participated in three uni-professional focus groups (6 clinical pharmacists, 6 physicians, and 7 nurses) and four one-to-one interviews with patient representatives: two patients recruited from the ED and two PFAC members. Four (three patient representatives and one ED physician) declined participation and did not provide a reason. Mean participant age was 40.8 year (range 30- 65 years), with nearly equal gender distribution (52% females).

#### **Phase II**

Included nine participants, four of whom had participated in Phase I (two clinical pharmacists, one nurse, and one PFAC member). New participants included a hospital executive and leaders from ED medicine, ED nursing, pharmacy, and the medical division, with some invitees nominating representatives because they were unable to attend. The majority were females (66.7), with a mean healthcare experience of 18 years (range 10–25 years).

### Deciding potential program users (eligibility criteria)

Stakeholders jointly refined eligibility criteria to prioritize high-risk patients while maintaining feasibility and efficiency within the ED context.

In Phase I, proposed inclusion criteria comprised older adults, polypharmacy (defined as taking ≥ 5 regular medications [51]), acute exacerbations of chronic illnesses, and chronic kidney disease (CKD); proposed exclusions, included poor adherence which was perceived as contributing to ED visits driven by behavioral rather than medical need, uncooperativeness, and logistical barriers to intervention delivery (e.g., transit passengers, visitors, or language barriers for non-Arabic/English speakers).

In Phase II, only criteria endorsed by at least two stakeholder groups were retained. Consensus resulted in restricting older adults to those meeting an additional inclusion criterion, retaining long-term visitors and poor adherence (noting the pharmacists' role in addressing the latter), removing CKD as a standalone criterion, and excluding patients with recurrent socially driven ED visits in the absence of acute medical condition.

Proposed inclusion and exclusion criteria and Phase II consensus decisions are presented in Table S4 (Supplementary file 1).

### Feasibility ratings and prioritization of program components

Medication reconciliation was rated the least feasible component (77.8%), primarily due to low practicability, whereas post-discharge follow-up received the highest feasibility rating (87.8%), driven by high acceptability and perceived effectiveness. Key implementation barriers included limited stakeholder awareness and acceptance of pharmacists’ ToC roles, concerns regarding discharge delays and increased workload associated with pharmacist involvement, competing ED priorities, care coordination challenges, patient-related barriers to medication reconciliation and education, and limited pharmacist awareness of community support resources. A more detailed qualitative analysis of the implementation barriers and facilitators identified in Phase I has been reported elsewhere [53].

Workshop participants reached broad consensus to retain all five proposed components, considering their feasibility and interdependence. The final co-designed intervention package comprised of five pharmacist-led components including medication history taking and discharge medication reconciliation, review of the discharge prescription, patient education and self-management support at discharge, discharge planning including structured handover and coordination of post-discharge follow-up, and pharmacist-led post-discharge review through a MTM clinic. These were supported by context-specific implementation resources including standardised workflows, documentation templates, patient education materials, and a clinical pharmacist training manual.

The APEASE feasibility ratings and supporting stakeholder quotes are presented in Table 1.

**Table 1:** APEASE feasibility grid: ratings and supporting stakeholder quotes^a,b^

Intervention	A	P	E	Af	S	E	Overall feasibility score (%)	Supporting Quotes
Medication history taking and discharge medication reconciliation	CP: 4 MD: 5 N: 3	CP: 2 MD: 3 N: 4	CP: 5 MD: 5 N: 5	CP: 3 MD: 5 N: 2	CP: 4 MD: 4 N: 3	CP: 5 MD: 5 N: 3	**77.8%**	Patient 1 “I think that people will accept it, they are educated, they know that this is very important, it's about their health” ED nurse 8: “Let me divide it into two categories. For budget and resources, I don’t know what you (pharmacy) have. But for return on investment, I give it a 5 because it directly touches patient safety.” ED physician 1″ For me it's 5 (effectiveness). It will have significant impact on practice”
Total score (%)	12 (80%)	9 (60%)	15 (100%)	10 (66.7%)	11 (73.3%)	13 (86.7%)		
Review of the discharge prescription	CP: 4 MD: 5 N: 5	CP: 3 MD: 3 N: 3	CP: 5 MD: 5 N: 5	CP: 3 MD: 5 N: 5	CP: 4 MD: 4 N: 3	CP: 5 MD: 5 N: 3	**83.3%**	Patient 3”Pharmacist knows better than anybody else about the drug. There will be no drug interactions, no fatal things would happen to the patients” CP 6 “If it's like the peak time and the physician has many patients to review, this might make them a bit reluctant to help in resolving medication issues”
Total (average)	14 (93.3%)	9 (60%)	15 (100%)	13 (86.7%)	11 (73.3%)	13 (86.7%)		
Patient education and self-management support at discharge	CP: 4 MD: 5 N: 5	CP: 2 MD: 3 N: 3	CP: 5 MD: 5 N: 5	CP: 3 MD: 5 N: 4	CP: 4 MD: 4 N: 4	5 MD: 5 N: 3	**82.2%**	Patient 2 “It’s acceptable to me. In fact, it’s what I am looking for. It’s not just about giving medicine and that’s it. No, you need to understand what is the purpose, and if specific symptoms appear you need to know how to take appropriate actions” ED nurse 4″ Maybe you will take time to reach the patient to explain about things” ED Physician 5 “For some patients the language barrier, you cannot understand their language, and for some languages we do not have enough staff to translate”
Total (average)	14 (93.3%)	8 (53.3%)	15 (100%)	12 (80%)	12 (80%)	13 (86.7%)		
Discharge planning, including timely coordination, structured hand-offs, and scheduling of post-discharge follow-up^c^	CP: 4	CP: 4	CP: 5	CP: 3	CP: 5	CP: 5	**86.6%**	CP 5 “It’s practical, having a template would even make it more easy. Things that should be followed up, doses that have been recently changed, compliance issue with certain medications, cost issues, unresolved problems that sometimes we don't have the time to resolve in ED”
Total (average)	4 (80%)	4 (80%)	5 (100%)	3 (60%)	5 (100%)	5 (100%)		
Post-discharge follow-up delivered through a pharmacist-led MTM clinic	CP: 4 MD: 5 N: 5	CP: 3 MD: 4 N: 3	CP: 5 MD: 5 N: 5	CP: 3 MD: 5 N: 3	CP: 5 MD: 4 N: 5	CP: 5 MD: 5 N: 5	**87.8%**	Patient 3″ I'll be comfortable. I'll be happy if the pharmacist calls me about my medicine, about the interactions, about my health, I'll appreciate it. I definitely do” Patient 2 "A phone call (consultation) might take only 15 min, whereas the trip to the hospital could take an hour and a half" ED Physician 2 “It takes a while for patients to get a schedule for follow up after discharge. So in between, somebody has to make sure that they're taking the correct medication, the correct way. It's beyond the emergency room scope. So, I think it will be very essential for our pharmacists to be that someone” CP 5 “Doctors might not really accept it because they might have a problem with how practical this is, like “OK, you follow up the patient in the clinic. Are you going to call me back to adjust any orders?” CP 4 “With current budget and staff, this increases their work”
Total (average)	14 (93.3%)	10 (66.7%)	15 (100%)	11 (73.3%)	14 (93.3%	15 (100%)		
Program evaluation (total)	89%	63%	100%	76%	82%	91%	83%	

*CP* clinical pharmacist, *MTM* medication therapy management. (A: Acceptability | P: Practicability | E: Effectiveness | Af: Affordability | S: Safety | E: Equity)

^**a**^Total scores were calculated by summing stakeholder ratings for each APEASE criterion (maximum = 15). Criterion percentages were calculated by dividing the total score by the maximum possible score. Overall feasibility scores (%) were calculated by dividing the total score across all six APEASE criteria by the maximum possible overall score (90). For the discharge planning intervention, only clinical pharmacists rated the intervention; therefore, the maximum possible score was adjusted accordingly (5 per criterion; 30 overall)

^**b**^Ratings were based on a 5-point Likert scale (1 = lowest; 5 = highest)

^c^Assessed by clinical pharmacists only

### Program specifications and workflow

Final program specifications and workflows reflected iterative refinement of Phase I proposals through Phase II co-design consensus and post-workshop operationalization.

**Fig. 2:**
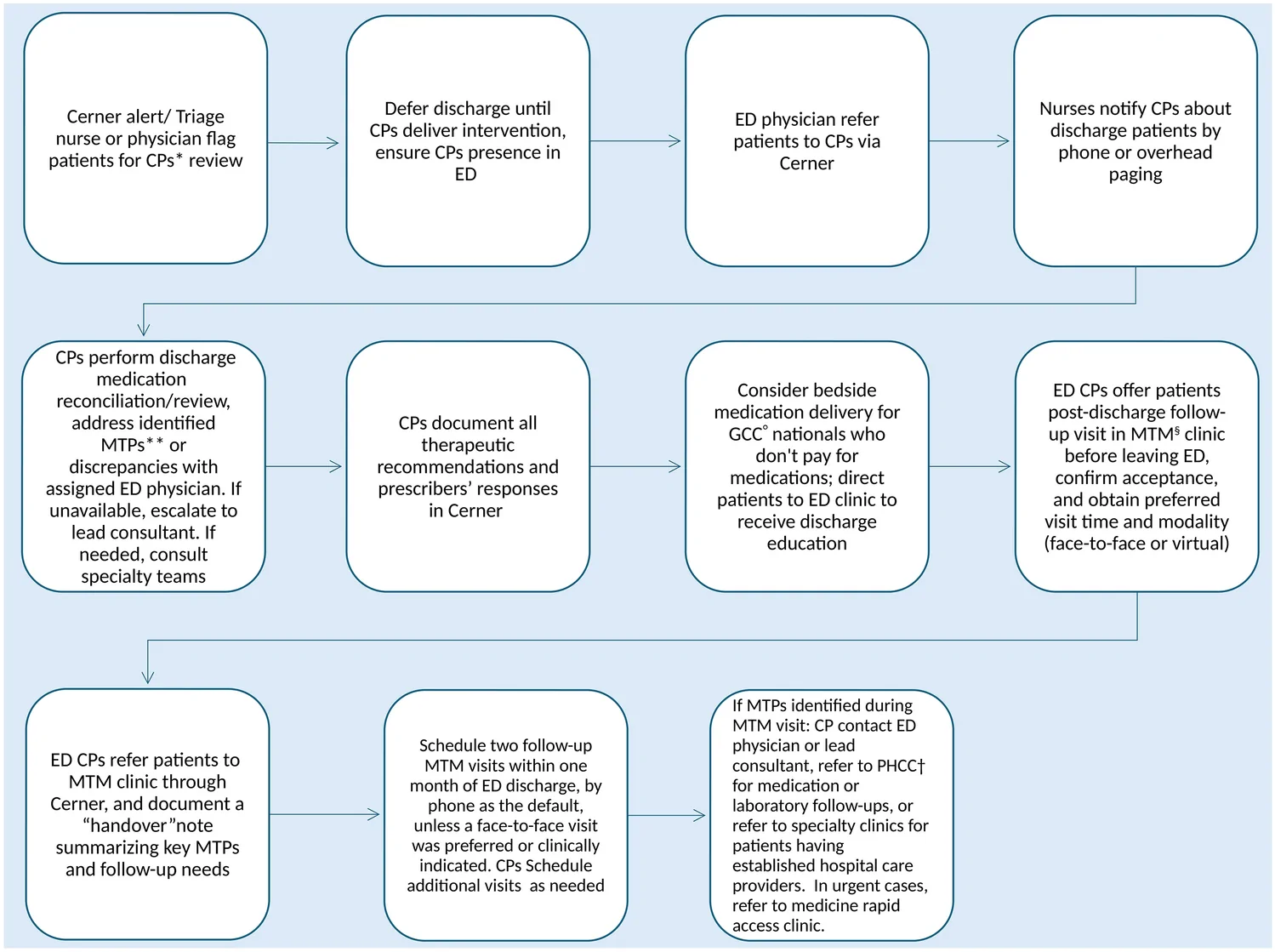
Phase I proposed workflow. Abbreviations: CMM: Comprehensive Medication Management; CP: Clinical Pharmacist; GCC: Gulf Cooperation Council; MTM: Medication Therapy Management; MTP: Medication Therapy Problems; PHCC: Primary Health Care Corporation

**Fig. 3:**
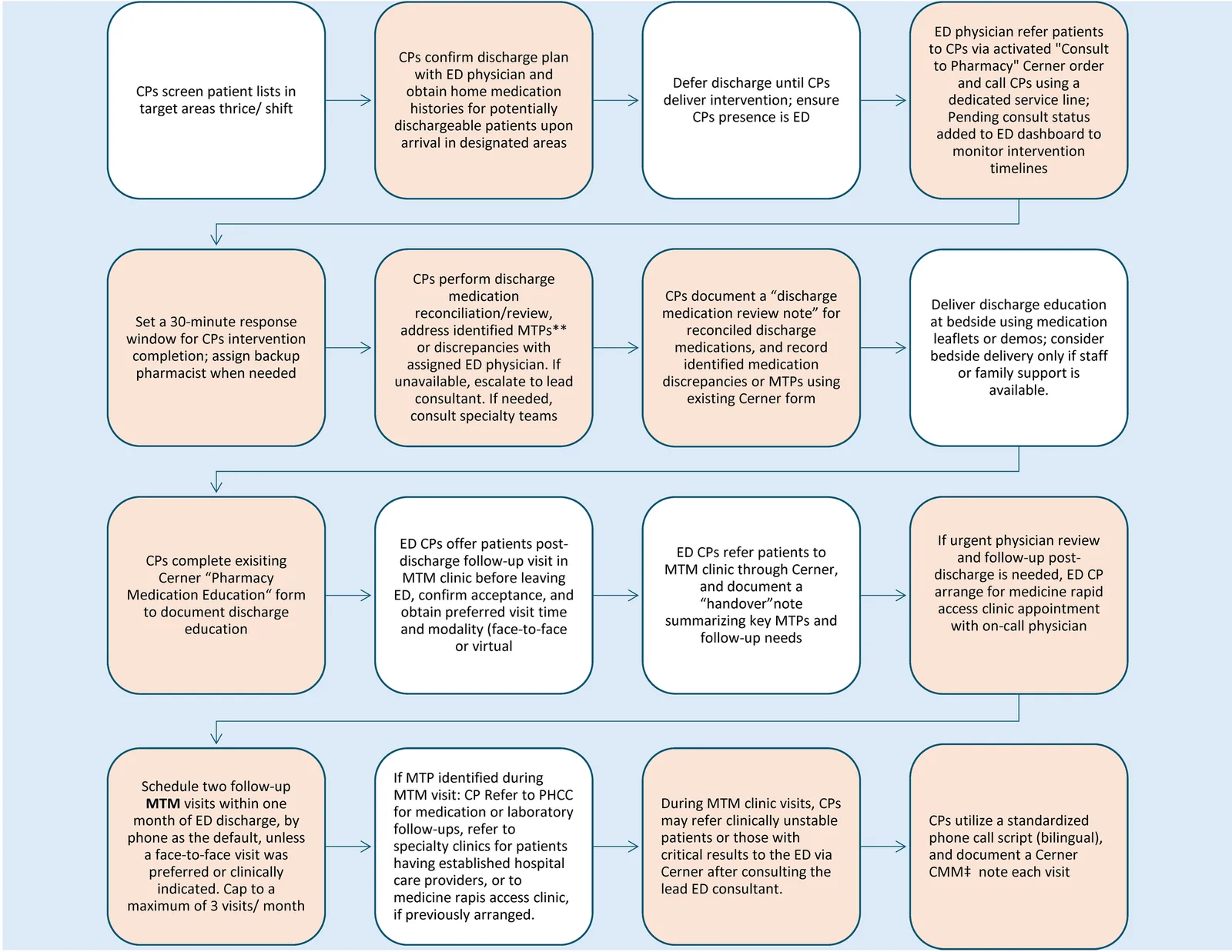
Final workflow of the Pharmacist-led intervention program. CMM: Comprehensive Medication Management; CP: Clinical Pharmacist; GCC: Gulf Cooperation Council; MTM: Medication Therapy Management; MTP: Medication Therapy Problems; PHCC: Primary Health Care Corporation Centers

Table 2 summarizes the development of each intervention component across these stages; detailed specifications and supporting stakeholder quotes are presented in Table S5 (Supplementary file 1). Evolving workflow models are illustrated in Fig. 2 (Phase I workflow) and Fig. 3 (final workflow).

**Table 2:** Iterative development of intervention components from Phase I proposals through Phase II co-design consensus to post-workshop operationalization

Intervention component	Phase I: stakeholder proposals and concerns	Phase II: co-design decisions	Post-Phase II follow-up: operationalized outputs
1. Discharge medication reconciliation and review	Limited ED clinical pharmacists' availability and workflow integration were identified as key practicability concerns, raising risks of discharge delays and missing eligible patients. Early patient identification and timely pharmacist notification were emphasized. Proposed solutions included electronic health record (EHR) alerts, triage-level eligibility screening by physicians or nurses, physician-initiated pharmacist referrals, deferral of discharge orders pending pharmacist intervention, and maintaining a dedicated pharmacist presence within the ED. Clinical pharmacists further highlighted the need for clear pathways to address medication issues identified during reconciliation and review, including documentation standards. Communication with the in-charge ED physician, with potential escalation to the lead consultant, and consultation of specialist teams when needed, was suggested	Triage-based patient flagging was discarded due to practical challenges. Clinical pharmacists were designated to proactively screen patients upon arrival to designated areas, review medication histories, and liaise with physicians at discharge. The existing “consult to pharmacy” Cerner order was endorsed for physician-initiated referrals, with a suggestion to integrate its status into the ED dashboard to track patients and intervention timelines. The proposed pharmacist–physician communication cascade was accepted, with consensus that clinical pharmacists must notify ED physicians of medication changes resulting from specialty consultations (e.g., cardiology)	The “consult to pharmacy” order was refined, activated, and its status integrated into the ED dashboard. Documentation processes were agreed upon with the clinical pharmacy team, including use of the “discharge medication review note” and existing Cerner-based documentation forms
2. Discharge medication counseling and education	Clinical pharmacists considered discharge counseling most effective when medications were physically available; however, difficulties navigating to the ED pharmacy to collect medications, and the restricted ED pharmacy formulary, were identified as key barriers. Proposed solutions included bedside medication delivery and the provision of education in a dedicated ED-based clinic, with patients given the option to choose their preferred location (i.e., bedside or clinic-based)	The stakeholders agreed on case-by-case bedside medication delivery, depending on staff or family availability, and endorsed the use of medication photos or demonstration devices (e.g., inhalers) as teaching aids when medications were unavailable. Consensus favored bedside over clinic-based delivery, citing greater practicability, patient-centeredness, and cultural alignment	The research and clinical pharmacy team co-designed an electronic library of patient education materials for common ED discharge medications, incorporating medication photos or demonstration videos. A sample leaflet was reviewed by a patient representative. Materials were made accessible via the MS Teams portal for printing or digital distribution (e.g., WhatsApp, email)
3. Planning post-discharge follow-up and handover communication	The stakeholders emphasized notifying patients about post-discharge follow-up prior to ED departure to support engagement. Proposed ED clinical pharmacist responsibilities included obtaining patient consent to attend the clinic, confirming preferred timing and modality (face-to-face or virtual), placing Cerner referrals to the MTM clinic, coordinating urgent reviews with the internal medicine rapid access clinic, and providing concise handoffs to ambulatory care clinical pharmacists focused on key medication therapy problems (MTPs)	The proposal was endorsed by all participants without modification	A standardized handover note template was co-designed with the clinical pharmacy team
4. Post-discharge follow-up with the pharmacist-led MTM clinic	A minimum of two follow-up visits within one month of ED discharge was proposed, with telephone consultation as the default modality unless a face-to-face visit was preferred by the patient or clinically indicated. To facilitate resolution of post-discharge MTPs, communication with ED physicians and referrals to the PHCC centers for medication or laboratory follow-up were proposed	The proposed pathway of communicating MTPs to the ED physicians was discarded due to concerns about workload and scope of practice. Instead, participants supported the existing practice of coordinating with previously assigned physicians within the same health system or PHCC centers. Ambulatory care clinical pharmacists were also advised to refer clinically unstable patients or those with critical results back to the ED following discussion with the lead consultant	Boston University’s Project RED phone call script was adapted through literature review and clinical pharmacy team meetings to standardise follow-up consultations [52], translated into Arabic by one clinical pharmacist, and validated by two others. The existing comprehensive medication management (CMM) note template was adopted for Cerner documentation after each visit

*CMM* Comprehensive Medication Management; *ED* Emergency Department; *EHR* Electronic Health Record; *MTM* Medication Therapy Management; *MTP* Medication Therapy Problem; *PHCC* Primary Health Care Corporation

### Process indicators for pilot evaluation

Guided by the UK’s Medical Research Council (MRC) framework for process evaluations (7), process indicators were predefined for future pilot evaluation using qualitative stakeholder interviews and quantitative trial implementation metrics **(**Figure S1, Supplementary file 1).

### Implementation strategies (program-level)

Barrier mapping prioritized six intervention functions: education, training, enablement, persuasion, incentivization, and environmental restructuring, yielding 31 corresponding implementation strategies, of which six were adopted. These comprised using media to encourage patients to bring medications to healthcare visits (education); creating communication channels among stakeholders and key leaders, providing standardization tools for clinical pharmacists delivering the intervention (e.g., a training manual, standardized documentation notes, and phone call scripts), and offering flexible follow-up appointment times and modalities according to patient preferences (enablement); conducting local consensus discussions involving key leaders in developing the program workflow (persuasion); and designing electronic templates for clinical pharmacists' documentation in Cerner (environmental restructuring).

Post-workshop operationalization established communication channels (MS Teams and WhatsApp groups) and orientation materials (PowerPoint presentations, handouts, and a CP training manual). A co-designed patient education leaflet advising patients to bring their medications to healthcare visits was adopted from an ongoing hospital initiative.

Detailed mapping of barriers to TDF and CTF domains, alongside corresponding SELECT and CFIR-ERIC implementation strategies, stakeholder-driven solutions, and final operationalized implementation strategies supporting overall program adoption and integration are presented in Table S6 (Supplementary file 1).

### Logic model development

**Fig. 4:**
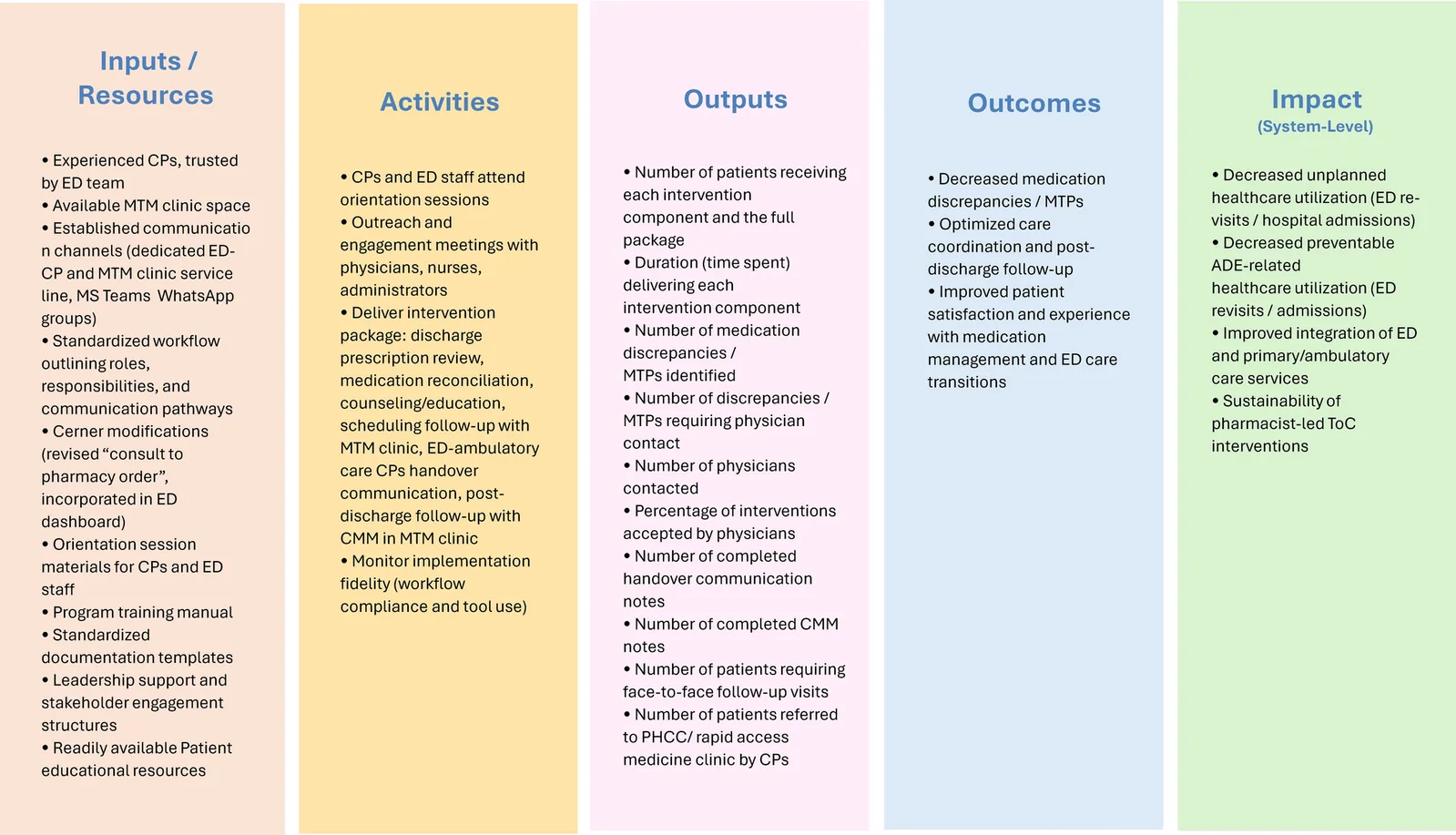
Logic model for a Pharmacist-led ED ToC program

A logic model providing a graphical/textual representation of the intervention’s proposed mechanism of impact, linking the program’s resources and processes with proposed outcomes was developed and presented in Fig. 4**.**

## Discussion

This study co-designed a multifaceted pharmacist-led ToC intervention in the ED, using a systematic, theory- and evidence-informed participatory approach that engaged key stakeholders to produce a clear blueprint of core components, delivery formats, workflow, and context-specific implementation strategies.

Intervention feasibility will be tested in a pilot RCT following UK Medical Research Council’s guidance for complex interventions [54], with a definitive RCT to follow if feasible.

The study addressed a key gap in ED ToC literature, the lack of transparent, stakeholder-informed intervention development [55, 56], that limits reproducibility and across-setting comparisons. While codesign approaches are increasingly employed in healthcare [27] and linked to improved outcomes [57], user satisfaction, and service efficiency [58], the diversity of codesign frameworks and models [27] presents challenges for methodological rigor, reproducibility, and comparability across studies [25, 59]. By integrating structured frameworks, TDF, CTF, APEASE, and COM-B, within a PHR paradigm, we aimed to balance theoretical grounding with flexibility needed for local adaptability, offering a pragmatic and reproducible model for ED intervention development. Further research is needed to clarify codesign frameworks and standardize their applications [27].

Another limitation of existing pharmacy-supported ToC literature, including ED, is the lack of well-defined, medication-related patient inclusion criteria [10, 60, 61], potentially diluting intervention effects, and explaining limited benefits. In contrast, we identified target users most likely to benefit from the intervention based on contextual needs and experiences, thus enhancing efficiency and addressing this ToC literature gap. Nonetheless, some exclusion criteria were introduced for operational feasibility, which may have ruled out patients at a particularly high risk of transition failure. While these decisions were necessary to support intervention delivery within the ED workflow, they may potentially reduce the equity, relevance, and generalizability of the proposed program.

Existing pharmacist-led ED ToC research has largely examined isolated intervention components such as medication reconciliation [62], discharge prescription review [63, 64], or pending microbiological culture follow-up [65] with limited attention to integrated, multi-component models that reflect the complexity of real-world transitions. While the optimal number and types of transitional care interventions remain uncertain, evidence suggests that a greater intensity of pharmacist-delivered components is associated with reduced healthcare utilization among older adults during care transitions [66]. Our co-designed intervention incorporates multiple components spanning pre-discharge, post-discharge follow-up, and the bridging period in between.

The APEASE criteria are increasingly used in pharmacy practice [67, 68] to evaluate and select intervention functions, modes of intervention delivery, policy categories, and behavior change techniques (BCTs).We applied APEASE directly to ToC intervention components, treated as a discrete intervention function within the Behavior Change Wheel framework, to support a pragmatic assessment of feasibility and contextual relevance. Completion of the APEASE grid was challenging due to subjective ratings, overlap in perceived facilitators and barriers across components, and the commonality in affordability and equity remarks leading to some redundancy. Equity ratings were particularly challenging because the language barrier was the only factor identified as contributing to inequity. Although some reported ToC challenges included financial difficulties, participants did not perceive these as creating inequitable access. This reflects the healthcare context in Qatar, where, at the time of writing, all nationals and expatriate residents are covered by the governmental health sector, with residents paying only nominal fees for services. Nevertheless, transitional care inequities may also arise from factors not specifically explored in this study, including racial sociocultural background, caregiver availability, transportation access, and broader social determinants that may influence patients’ adherence to post-discharge instructions and ability to attend post-discharge follow-up care [69, 70].

### Strengths and limitations

A key strength of this study is integrating CTF with TDF, for a comprehensive view of organizational and contextual factors in care transitions, addressing TDF’s limited organizational focus [71]. This approach, applied previously in implementation science [72, 73], improves the ability to capture the complexity of healthcare interventions. Nonetheless, combining multiple theoretical frameworks can increase methodological complexity and the potential for conceptual overlap [74, 75]. To mitigate this, the rationale and distinct role of each framework were specified a priori, and data were analyzed both inductively and deductively, allowing findings beyond the selected frameworks to emerge.

Additionally, the active engagement of diverse stakeholders as genuine partners throughout the codesign process, rather than passive feedback providers, represents a key strength of this study.

Nonetheless, one limitation of this study is the absence of a formal framework to assess stakeholder engagement [76]. However, engagement was supported through tailored interview guides, separate patient and practitioner sessions, and diverse methods including democratic dialogue, brainstorming, and structured voting. Phase I included a relatively small sample, particularly only four patient representatives, two of whom were PFAC members. This may have limited the breadth of perspectives and introduced “super-user” bias [77]. However, all patient representatives had prior ED discharge experience, and the consistency of views reported suggested adequate information power. Consensus-based discussions may have been influenced by group dynamics, social desirability, and the first author's (EH) managerial role at the study site. The study also included only Arabic- and English-speaking participants, limiting the representation of patients from other language backgrounds. Only one codesign workshop was conducted due to work constraints, a challenge commonly reported in codesign studies [78, 79], although additional meetings, individual interviews, and email communications were used to finalize the intervention. Finally, as the intervention was co-designed in a single acute care setting, its transferability to other healthcare contexts needs careful examination.

## Conclusion

Underpinned by participatory codesign principles, theoretical frameworks, and stakeholder lived experiences, this study developed a contextually tailored pharmacist-led ToC program for patients discharged from ED. The study produced a structured multifaceted intervention, medication risk-based eligibility criteria, and implementation resources that support the program adoption and integration into ED workflow. By integrating operational, clinical, and patient perspectives, this work addresses an important gap in ED pharmacy ToC literature, where contextually developed interventions remain limited. Future research should evaluate the program’s feasibility, implementation, and impact on patient care.

## Supplementary Information

Below is the link to the electronic supplementary material.

Supplementary file1 (DOCX 737 KB)

Supplementary file2 (DOCX 44 KB)

## Data Availability

The datasets generated and/or analyzed during the current study are not publicly available due to participant confidentiality considerations but are available from the corresponding author upon reasonable request.
