# Supplementary material for: Codesigning a pharmacist-led transition of care program in the emergency department: a qualitative participatory study
Source: Int J Clin Pharm. 2026 Aug 14;48(6):2322–38. doi: 10.1007/s11096-026-02210-3 (PMC13731452; doi:10.1007/s11096-026-02210-3)
Supplement: Supplementary file 1 [file 11096_2026_2210_MOESM1_ESM.docx]

**Supplementary File 1**

**Title:** Codesigning a pharmacist-led transition of care program in the emergency department: A qualitative participatory study

**Journal:** International Journal of Clinical Pharmacy (IJCP)

**Authors:** Authors: Eman Al Hmoud(1,2), Raja Barazi(2), Binny Thomas(2), Almunzer Zakaria(2), Lubna Andraous(2), Rasha El Enany(2), Palli Valapila Abdulrouf, Sameer Pathan(2), Hamde Nazar(3), Mohamed Izham Mohamed Ibrahim(4), *Muhammad Abdul Hadi(4)

**Authors’ Affiliations:**

1.	QU Health Sector, Qatar University, Doha, Qatar

2.	Hamad Medical Corporation, Doha, Qatar

3.	National Institute for Health Research (NIHR) Newcastle Patient Safety Research Collaboration, Newcastle University, Newcastle-upon-Tyne, United Kingdom

4. College of Pharmacy, QU Health, Qatar University, Doha, Qatar

***Corresponding author**

Muhammad Abdul Hadi

Professor of Clinical Pharmacy and Practice

Graduate Coordinator: PhD in Health Sciences (Clinical and Population Health Research)

Department of Clinical Pharmacy and Practice

College of Pharmacy, QU Health,

Qatar University

Doha, P.O. Box 2713

Qatar

Tel: 00974 4403 5582

Email: [mabdulhadi@qu.edu.qa](mailto:mabdulhadi@qu.edu.qa)

**Supplementary Tables and figures**

| **Table** | **Page #** |
| --- | --- |
| **Table S1.** Guidance for reporting intervention development studies in health research (GUIDED) [1] |  |
| **Table S2.** COREQ (COnsolidated criteria for REporting Qualitative research) Checklist |  |
| **Table S3.** Stakeholder analysis using the influence/ interest grid |  |
| **Table S4.** Phase I proposed eligibility criteria and Phase II consensus decisions |  |
| **Table S5.** Intervention component iterations, workflow decisions, and final outputs |  |
| **Table S6.** Mapping implementation barriers to implementation support strategies |  |
| **Figure S1.** Key process evaluation indicators |  |

**Table S1. Guidance for reporting intervention development studies in health research (GUIDED) [1]**

| **Item description** | **Explanation** | **Page in manuscript where item is located** | **Other*** |
| --- | --- | --- | --- |
| 1. Report the context for which the intervention was developed. | Understanding the context in which an intervention was developed informs readers about the suitability and transferability of the intervention to the context in which they are considering evaluating, adapting or using the intervention. Context here can include place, organisational and wider sociopolitical factors that may influence the development and/or delivery of the intervention (15). | 5-6 | Study setting  The study was conducted at an 85-bed ED within a general teaching hospital under Hamad Medical Corporation (HMC), Qatar's primary public healthcare provider. Th ED serves approximately 161,000 patients annually and is supported by a single clinical pharmacist primarily covering critical care areas. The hospital operates the Cerner® electronic health record system, integrated across HMC and the Primary Health Care Corporation (PHCC) centers, and hosts a full-time pharmacist-led medication therapy management (MTM) clinic. |
| 2. Report the purpose of the intervention development process. | Clearly describing the purpose of the intervention specifies what it sets out to achieve. The purpose may be informed by research priorities, for example those identified in systematic reviews, evidence gaps set out in practice guidance such as The National Institute for Health and Care Excellence or specific prioritisation exercises such as those undertaken with patients and practitioners through the James Lind Alliance. | 1 (Abstract); 5 (Aim; Study design) | (Background and Design)  To co-design a pharmacist-led ToC programme improving medication management for adults discharged from the ED, addressing the gap in transparent, stakeholder-informed intervention development in the ED ToC literature, with subsequent evaluation planned in a pilot RCT. |
| 3. Report the target population for the intervention development process. | The target population is the population that will potentially benefit from the intervention – this may include patients, clinicians, and/or members of the public. If the target population is clearly described then readers will be able to understand the relevance of the intervention to their own research or practice. Health inequalities, gender and ethnicity are features of the target population that may be relevant to intervention development processes. | 11-12; Supplementary file 1, Table S4 |  |
| 4. Report how any published intervention development approach contributed to the development process | Many formal intervention development approaches exist and are used to guide the intervention development process (e.g. 6Squid (16) or The Person Based Approach to Intervention Development (17)). Where a formal intervention development approach is used, it is helpful to describe the process that was followed, including any deviations. More general approaches to intervention development also exist and have been categorised as follows (3):- Target Population-centred intervention development; evidence and theory-based intervention development; partnership intervention development; implementation-based intervention development; efficacybased intervention development; step or phased-based intervention development; and intervention-specific intervention development (3). These approaches do not always have specific guidance that describe their use. Nevertheless, it is helpful to give a rich description of how any published approach was operationalised | 5 (Study design); 7-9 (Data collection and analysis) | A two-phase qualitative study within a Participatory Health Research (PHR) framework [35], guided by co-design principles of inclusivity, participation, equity, iterative refinement, and transparency [36]. Reflects both evidence- and theory-based and partnership-based intervention development. Phase I used inductive-deductive qualitative analysis guided by TDF and CTF. Phase II used a structured co-design workshop and electronic prioritization survey, followed by post-workshop operationalization. Implementation strategy mapping used the SELECT [48] and CFIR-ERIC [49] tools. Reported in accordance with GUIDED [37]. |
| 5. Report how evidence from different sources informed the intervention development process. | Intervention development is often based on published evidence and/or primary data that has been collected to inform the intervention development process. It is useful to describe and reference all forms of evidence and data that have informed the development of the intervention because evidence bases can change rapidly, and to explain the manner in which the evidence and/or data was used. Understanding what evidence was and was not available at the time of intervention development can help readers to assess transferability to their current situation. | 5, 7 | Five proposed components were derived from a prior systematic review [41] and professional guidelines [42, 43]. Feasibility was assessed using the APEASE criteria during Phase I. Implementation barriers were mapped to intervention functions and strategies using SELECT [48] and CFIR-ERIC [49]. Stakeholder-proposed workflow solutions and post-workshop CP discussions further informed the evidence base. The post-discharge follow-up script was adapted from Boston University's Project RED. |
| 6.Report how/if published theory informed the intervention development process. | Reporting whether and how theory informed the intervention development process aids the reader’s understanding of the theoretical rationale that underpins the intervention. Though not mentioned in the e-Delphi or consensus meeting, it became increasingly apparent through the development of our guidance that this theory item could relate to either existing published theory or programme theory | 5-7 | Four frameworks informed the study: TDF [39] structured individual-level determinant analysis; CTF [40] guided organizational and contextual analysis of care transitions; COM-B (via SELECT) [48] underpinned implementation strategy selection; and the Behaviour Change Wheel/APEASE [44, 45] guided feasibility evaluation and intervention function selection. CFIR (parent framework of CTF) [49] linked identified barriers to ERIC implementation strategies. |
| 7. Report any use of components from an existing intervention in the current intervention development process. | Some interventions are developed with components that have been adopted from existing interventions. Clearly identifying components that have been adopted or adapted and acknowledging their original source helps the reader to understand and distinguish between the novel and adopted components of the new intervention. | 7, 25 | Five components adopted and contextually adapted from the systematic review [41] and guidelines [42, 43]: (1) medication history taking and discharge reconciliation; (2) discharge prescription review; (3) patient education and self-management support; (4) discharge planning, structured hand-offs, and post-discharge appointment coordination; (5) pharmacist-led post-discharge follow-up in the MTM clinic. Boston University's Project RED phone call script was adapted, translated into Arabic, and validated by the CP team for component 5. |
| 8. Report any guiding principles, people or factors that were prioritised when making decisions during the intervention development process. | Reporting any guiding principles that governed the development of the application helps the reader to understand the authors’ reasoning behind the decisions that were made. These could include the examples of particular populations who views are being considered when designing the intervention, the modality that is viewed as being most appropriate, design features considered important for the target population, or the potential for the intervention to be scaled up. | 5, 11-19 | Co-design principles of inclusivity, participation, equity, iterative refinement, and transparency guided all phases. Stakeholder mapping using the influence-benefit grid prioritized those with high influence and benefit in ED ToC. APEASE ensured contextual relevance and equity were embedded in all component decisions. Patient-centeredness and cultural alignment (e.g., bedside counselling preference) were consistently prioritized. Consensus was defined a unanimous agreement. Implementation strategies selected based on 100% agreement. |
| 9. Report how  stakeholders  contributed to the  intervention  development process | Potential stakeholders can include patient and community representatives,  local and national policy makers, health care providers and those paying for or  commissioning health care. Each of these groups may influence the intervention development process in different ways. Specifying how differing groups of stakeholders contributed to the intervention development process  helps the reader to understand how stakeholders were involved and the degree of influence they had on the overall process. Further detail on how to integrate stakeholder contributions within intervention reporting are available (19). | 6-8 (Participants; Data collection); 11-19 (Results) | Patients/representatives, clinical pharmacists, physicians, and nurses identified barriers and appraised components (Phase I); decision makers, departmental leaders, and Phase I representatives co-designed eligibility criteria, workflows, and implementation strategies (Phase II workshop); post-workshop operationalization with the clinical pharmacy team; prototype and logic model disseminated to key stakeholders. |
| 10. Report how the  intervention changed in  content and format  from the start of the  intervention  development process. | Intervention development is frequently an iterative process. The conclusion  of the initial phase of intervention development does not necessarily mean  that all uncertainties have been addressed. It is helpful to list remaining  uncertainties such as the intervention intensity, mode of delivery, materials,  procedures, or type of location that the intervention is most suitable for. This  can guide other researchers to potential future areas of research and  practitioners about uncertainties relevant to their healthcare context. | 11-12, 16-21 | The intervention evolved iteratively across three stages |
| 11. Report any changes to interventions required or likely to be required for subgroups | Specifying any changes that the intervention development team perceive are required for the intervention to be delivered or tailored to specific subgroups enables readers to understand the applicability of the intervention to their target population or context. These changes could include changes to personnel delivering the intervention, to the content of the intervention, or to the mode of delivery of the intervention. | NA | No subgroup-specific changes were identified. Language accessibility was addressed through Arabic translation of patient education materials and the post-discharge script. Non-Arabic/English-speaking patients were not enrolled; this limitation is acknowledged in the manuscript. |
| 12.Report important uncertainties at the end of the intervention development process. | Intervention development is frequently an iterative process. The conclusion of the initial phase of intervention development does not necessarily mean that all uncertainties have been addressed. It is helpful to list remaining uncertainties such as the intervention intensity, mode of delivery, materials, procedures, or type of location that the intervention is most suitable for. This can guide other researchers to potential future areas of research and practitioners about uncertainties relevant to their healthcare context. | 25-27 | - *Acknowledged in Discussion and Conclusions* Remaining uncertainties include optimal post-discharge follow-up intensity and frequency; scalability beyond the single-pharmacist ED model; generalizability to other language groups; and long-term sustainability of implementation strategies. These will be addressed in the planned pilot RCT and process evaluation per MRC framework guidance . |
| 13.Follow TIDieR guidance when describing the developed intervention | Interventions have been poorly reported for a number of years. In response to this, internationally recognized guidance has been published to support the high quality reporting of health care? interventions^5^and public health interventions^14^. This guidance should therefore be followed when describing a developed intervention. | 11-12, 16-24 | Intervention components, rationale, delivery format, provider, setting, workflow, and implementation materials are described across the results section and supplementary files. The logic model (Figure 4) provides a graphical representation of the program's proposed mechanism of impact. |
| 14. Report the intervention development process in an open access format | Unless reports of intervention development are available people considering using an intervention cannot understand the process that was undertaken and make a judgement about its appropriateness to their context. It also limits cumulative learning about intervention development methodology and observed consequences at later evaluation, translation and implementation stages. Reporting intervention development in an open access (Gold or Green) publishing format increases the accessibility and visibility of intervention development research and makes it more likely to be read and used. Potential platforms for open access publication of intervention development include open access journal publications, freely accessible funder reports or a study web-page that details the intervention development process. | NA | Submitted to International Journal of Clinical Pharmacy. Study protocol published open access in BMJ Open. |

*e.g. if item is reported elsewhere, then the location of this information can be stated here.

**Table S2. COREQ (COnsolidated criteria for REporting Qualitative research) Checklist** [2]

| **Topic** | **Item No.** | **Guide Questions/Description** | **Reported on Page No.** |
| --- | --- | --- | --- |
| **Domain 1: Research team and reflexivity** |  |  |  |
| *Personal characteristics* |  |  |  |
| Interviewer/facilitator | 1 | Which author/s conducted the interview or focus group? | Page 7 (interviews conducted by EH; focus groups moderated by EH with RB or MAH as note-takers); page 8 (workshop facilitated by EH, MAH note-taker) |
| Credentials | 2 | What were the researcher’s credentials? E.g. PhD, MD | Page 1 (author affiliations); page 7 |
| Occupation | 3 | What was their occupation at the time of the study? | Page 7 (EH: pharmacy leader at the study site; RB: clinical pharmacist; MAH: Professor of Clinical Pharmacy and Practice) |
| Gender | 4 | Was the researcher male or female? | Page 7 |
| Experience and training | 5 | What experience or training did the researcher have? | Page 7 (all research team members had qualitative research experience) |
| *Relationship with participants* |  |  |  |
| Relationship established | 6 | Was a relationship established prior to study commencement? | Page 7 (EH was acquainted with the clinical pharmacists but not with other stakeholders) |
| Participant knowledge of the interviewer | 7 | What did the participants know about the researcher? e.g. personal goals, reasons for doing the research | Pages 7, 27 (EH's role at the study site known to professional participants; addressed under limitations) |
| Interviewer characteristics | 8 | What characteristics were reported about the interviewer/facilitator? e.g. Bias, assumptions, reasons and interests in the research topic | Pages 9, 27 (reflexive discussions; potential social desirability and power imbalance acknowledged in limitations) |
| **Domain 2: Study design** |  |  |  |
| *Theoretical framework* |  |  |  |
| Methodological orientation and Theory | 9 | What methodological orientation was stated to underpin the study? e.g.  grounded theory, discourse analysis, ethnography, phenomenology, content analysis | Pages 5-6, 9 (participatory health research framework; theoretical underpinning by TDF and CTF; inductive thematic analysis [Braun & Clarke] and deductive framework analysis) |
| *Participant selection* |  |  |  |
| Sampling | 10 | How were participants selected? e.g. purposive, convenience, consecutive, snowball | Page 6 (purposive and snowball sampling guided by stakeholder influence-benefit mapping) |
| Method of approach | 11 | How were participants approached? e.g. face-to-face, telephone, mail, email | Page 6 (patients approached in person during ED visit or by telephone within one week of discharge; PFAC members by phone; healthcare professionals by email) |
| Sample size | 12 | How many participants were in the study? | Pages 6, 11 (information power; n=23 Phase I, n=9 Phase II) |
| Non-participation | 13 | How many people refused to participate or dropped out? Reasons? | Page 11 (four declined participation; no reasons provided) |
| *Setting* |  |  |  |
| Setting of data collection | 14 | Where was the data collected? e.g. home, clinic, workplace | Pages 7-8 (uni-professional focus groups and one-to-one interviews; two-hour in-person co-design workshop at the study site) |
| Presence of nonparticipants | 15 | Was anyone else present besides the participants and researchers? | Page 7 (note-takers RB or MAH present during focus groups; no other non-participants) |
| Description of sample | 16 | What are the important characteristics of the sample? e.g. demographic data, date | Page 11 (age, gender, professional group, healthcare experience) |
| *Data collection* |  |  |  |
| Interview guide | 17 | Were questions, prompts, guides provided by the authors? Was it pilot tested? | Page 7 (stakeholder-specific topic guides informed by TDF and CTF; published with the study protocol and provided in Supplementary file 2) |
| Repeat interviews | 18 | Were repeat interviews carried out? If yes, how many? | N/A (no repeat interviews conducted) |
| Audio/visual recording | 19 | Did the research use audio or visual recording to collect the data? | Pages 8-9 (all sessions audio-recorded) |
| Field notes | 20 | Were field notes made during and/or after the interview or focus group? | Page 8 (detailed field notes taken during the workshop) |
| Duration | 21 | What was the duration of the interviews or focus group? | Page 8 (workshop: two hours; interview/focus group duration not reported) |
| Data saturation | 22 | Was data saturation discussed? | Page 6 (sample size guided by information power rather than data saturation) |
| Transcripts returned | 23 | Were transcripts returned to participants for comment and/or correction? | N/A (transcripts not returned to participants) |
| **Topic** | **Item No.** | **Guide Questions/Description** |  |
| **Domain 3: analysis and findings** |  |  |  |
| *Data analysis* |  |  |  |
| Number of data coders | 24 | How many data coders coded the data? | Page 9 (two independent coders: EH, RB) |
| Description of the coding tree | 25 | Did authors provide a description of the coding tree? | Page 9 (deductive framework matrix mapping responses to TDF and CTF domains; Supplementary file 1, Table S5) |
| Derivation of themes | 26 | Were themes identified in advance or derived from the data? | Pages 9, 12 (inductive and deductive derivation; discrepancies resolved by consensus; inductively generated themes reported separately) |
| Software | 27 | What software, if applicable, was used to manage the data? | N/A (not reported) |
| Participant checking | 28 | Did participants provide feedback on the findings? | Page 9 (member checking undertaken by sharing Phase I summary findings with all 23 participants; no corrections or alternative interpretations were submitted; prototype intervention and logic model disseminated to key stakeholders) |
| *Reporting* |  |  |  |
| Quotations presented | 29 | Were participant quotations presented to illustrate the themes/findings?  Was each quotation identified? e.g. participant number | Pages 13-15 (Table 1: supporting stakeholder quotes identified by participant role); Supplementary file 1, Table S4 |
| Data and findings consistent | 30 | Was there consistency between the data presented and the findings? | Pages 11-25 (Results), 25-27 (Discussion) |
| Clarity of major themes | 31 | Were major themes clearly presented in the findings? | Pages 11-25 (results organized by co-design outputs: eligibility criteria, feasibility ratings, component specifications, implementation strategies) |
| Clarity of minor themes | 32 | Is there a description of diverse cases or discussion of minor themes? | Pages 11-12 (divergent stakeholder views reported, e.g., Phase I/Phase II positions on poor adherence) |

Developed from: Tong A, Sainsbury P, Craig J. Consolidated criteria for reporting qualitative research (COREQ): a 32-item checklist for interviews and focus groups. *International Journal for Quality in Health Care*. 2007. Volume 19, Number 6: pp. 349 – 357

**Table S3. Stakeholder analysis using the influence/ interest grid**

|  | **Low Interest** | **High Interest** |
| --- | --- | --- |
| **High Influence** | - Medical Director / Chief Executive Officer (CEO) - Hospital Board (Assistant Executive Directors – Non-Clinical Services) - Information Technology Leadership (including Pharmacy Informatics / Physician Cerner Team) - HMC Corporate Administrators / Policy Makers (outside AWH) - Finance Officers - Media / Communication Department (for approval and distribution of patient education materials) | - Emergency Department (ED) Leadership (ED Head, ED Nursing Director) - Pharmacy Leadership (Director of Pharmacy) - Medicine Department Leadership (Chairman of Medicine) - Assistant Executive Director (Clinical Services) - ED Physicians (Consultants) - ED Nurses (Head Nurses, Charge Nurses, Staff Nurses) - Medicine Department Physicians (Consultants, Specialists) - ED Clinical Pharmacists and Ambulatory Medication Therapy Management (MTM) Clinic Pharmacists (Program Implementers) - Patients or Caregivers - Patient and Family Advisory Council (PFAC) Members (highly motivated; strong voice in patient-centered projects) |
| **Low Influence** | - Non-Clinical Hospital Support Services (e.g., clerical staff) - Other Subspecialties (e.g., Surgery) - Academic Partners - Emergency Pharmacy Staff (Supervisor, Staff) - Primary Healthcare Corporation Centers (PHCC) Physicians | - Social Workers - Case Managers |

**Table S4. Phase I proposed eligibility criteria and Phase II consensus decisions**

| **Inclusion Criteria** | **Phase I Proposals**  **Suggested by:** | | | | | **Retained in Phase II (Y/N)** | **Rationale** |
| --- | --- | --- | --- | --- | --- | --- | --- |
|  | Pt. | MD | N | CP | SUM |  |  |
| Newly initiated high-risk medications at discharge (e.g., corticosteroids, antiepileptics, anticoagulants, insulin) | ✓ | ✓ | ✓ | ✓ | 4 | Y |  |
| Newly diagnosed chronic conditions (e.g., hypertension, diabetes, seizure disorder) | ✓ |  | ✓ | ✓ | 3 | Y |  |
| Polypharmacy | ✓ |  | ✓ | ✓ | 3 | Y |  |
| Exacerbation of chronic illnesses (e.g., bronchial asthma, chronic obstructive pulmonary disease, heart failure, inflammatory bowel diseases) |  | ✓ | ✓ |  | 2 | Y |  |
| Patients with reported drug-allergy (sometime falsely reported) |  | ✓ | ✓ |  | 2 | N | Not a reliable indicator of high medication-related risk |
| Elderly patients | ✓ | ✓ |  |  | 2 | N | Inclusion limited to older adults with ≥1 additional criterion to enhance efficiency |
| Chronic kidney disease patients | ✓ |  | ✓ |  | 2 | N | Disease heterogeneity, early-stage patients often not on medication and dialysis patients are closely monitored. |
| Transplant patients |  | ✓ |  |  | 1 | N | Low stakeholder agreement (low voting scores) in Phase I |
| Cancer patients |  | ✓ |  |  | 1 | N | Low stakeholder agreement (low voting scores) in Phase I |
| Recurrent ED visits |  |  | ✓ |  | 1 | N | Low stakeholder agreement (low voting scores) in Phase I |
| Patients with known poor adherence to medications |  |  |  | ✓ | 1 | N | Low stakeholder agreement (low voting scores) in Phase I |
| **Exclusion Criteria** | | | | | | | |
| Transit passengers |  | ✓ | ✓ | ✓ | 3 | Y |  |
| Visitors |  | ✓ | ✓ | ✓ | 3 | N  (If post-discharge length of stay in Qatar is less than 30 days) | Exclude only if length of stay post ED discharge <1 month, some visitors stay for several months and are covered by insurance |
| Patients presenting with acute minor illnesses | ✓ | ✓ | ✓ |  | 3 | Y |  |
| Lack of decision-making capacity (e.g., elderly, bed-ridden patients who come without a caregiver, difficult to take any history or talk to them, psychiatric patients, unstable psychiatric patients) |  | ✓ |  | ✓ | 2 | Y |  |
| Language barrier (non-Arabic/English speakers) |  |  | ✓ | ✓ | 2 | Y |  |
| Terminally ill patients |  | ✓ |  |  | 1 | Y |  |
| Patients with active substance abuse |  | ✓ |  |  | 1 | Y |  |
| Prisoners |  | ✓ |  |  | 1 | Y |  |
| Patients who have history of poor adherence to medications and known to be uncooperative with healthcare providers |  | ✓ |  |  | 1 | N | Pharmacists’ potential role in addressing adherence. It was decided to exclude patients with frequent ED visits for social reasons (no identifiable acute medical condition, already known to the ED team as recurrent visitors) |

Abbreviations: CP: Clinical Pharmacist; MD: Physician; N: Nurse; Pt: Patient, Y: Yes; N: No

** Polypharmacy: the following definition was adopted by the research team: patients taking ≥5 regular medications at the time of ED discharge [3]

**Table S5. Intervention component iterations, workflow decisions, and final outputs**

| **Intervention Component: Discharge Medication Reconciliation and Review (by CPs)** | | | | |
| --- | --- | --- | --- | --- |
| **Workflow** | **Phase I Suggestions** | **Phase II (Co-design Workshop) Refinements** | **Post-Workshop Operational Integration** | **Final Output** |
|  | - **Patient identification:**   Use Cerner alerts and early triage screening (by nurses or physicians) to flag eligible dischargeable patients.   - **Referral to CPs:**   ED physicians initiate referrals via Cerner; nurses notify CPs by phone or overhead paging.   - **Preventing premature discharge** (before intervention)**:** Defer discharge until CPs review, ensure CPs presence in ED. - **Communication and escalation pathways:**   CPs address identified medication therapy problems (MTPs) or discrepancies with the assigned ED physician. If unavailable, escalate to the lead consultant. If needed consult specialty teams (e.g., cardiology).   - **Documentation:**   CPs document all therapeutic recommendations and prescribers’ responses in Cerner in accordance with current practice. | - **Patient identification:** - Triage-based flagging discarded by ED staff due to practical challenges (e.g., brief encounter); Cerner modifications unfeasible. - CPs screen patient lists in target areas (to identify dischargeable patients) thrice/ shift, aligning with typical 2–3-hour length of stay and maximizing capture. - **Timing of pharmacist involvement:**   CPs discuss discharge plan with ED physician once patients arrive to target areas, obtain home medication history for potentially dischargeable patients (rather than waiting until discharge decision), allowing more time for comprehensive medication review.   - **Referral to CPs:**   ED physicians initiate referrals via Cerner using “Consult to Pharmacy” order and contact CPs’ through a dedicated service line.   - **Preventing premature discharge:**   Defer discharge until pharmacist review; ensure CPs presence in ED.   - **Minimizing discharge delays:** Set a 30-minute response window for CPs consults and assign a backup CP when needed. - **Documentation and tracking:** Incorporate a “pending consult” status in ED dashboard to monitor intervention timelines - **Communication and escalation pathways:** - The proposal was endorsed by all participants. - ED team emphasized that medication changes resulting from CPs’ consultations with specialty teams must be communicated to ED physicians, who hold ultimate discharge responsibility. | - **Revision of Cerner Consult Order:** - Senior CPs revised exiting previously inactive “Consult to Pharmacy” order to include new options (e.g., medication counseling and education, review of medication history, review of discharge medications) and priority levels (urgent: within 30-60 minutes). - The proposed changes were approved by the Corporate Pharmacy Informatics Working Group. - Revised consult order was integrated in Cerner and tested by CPs prior to implementation. - **Documentation and tracking:**   Agreement with the CP team that CPs will document a “discharge medication review note” for reconciled discharge medications, consistent with current practice for medicine-discharged patients, and record identified medication discrepancies or MTPs using the existing Cerner “Clinical Pharmacy Intervention” ad-hoc form. | - “Consult to Pharmacy” Cerner Order updated and added to real time ED Dashboard. |
| **Supporting Quotes** | *“Notification of the clinical pharmacist once patients from the target group arrive, like having someone from the triage nurses to call the pharmacist.”* (CP 6)  *“Referral. Like sometimes patient will be discharged, doctor writes referral for medicine, so it would be consultation for pharmacy”* (N 5)  *“Sometimes you find issues when reviewing medications not related to the acute visit and the ED physician is not a decision maker here. So, I suggest calling the on call cardiologist or nephrologist for example to sort this out”* (CP 5) | *“It’s not applicable for the triage physician or nurse. It's a big challenge. They will not be able to identify the patients. The place is very small. They are not doing thorough assessment, just a brief assessment to see what the priority is and move (patient) to another area” (ED Lead)*  *“Instead of waiting for the physician to place the discharge order and then initiate the pharmacist intervention, you can start from the previous step” (Hospital Executive)* |  |  |
| **Touch point: Discharge Medication Counseling and Education** | | | | |
|  | **Phase I Suggestions** | **Phase II (Co-design Workshop) Refinements** | **Post-Workshop Operational Integration** | **Final Output** |
| **Workflow** | - **Bedside medication delivery:** Was suggested, particularly for Gulf Cooperation Council (GCC) nationals who are exempt from medication payment. - **Relocating education to a dedicated space** (e.g., an ED- clinic), if feasible and acceptable by patients.   . | - **Bedside medication delivery:** Infeasible due to lack of dedicated staff, but could be considered on a case-by-case basis, depending on the availability of staff or family members. - **Relocating education to a dedicated space** (e.g., an ED- clinic) was discarded, with a broad consensus in favor of bedside education, being more practical, patient-centered, and culturally appropriate. - **Alternative Educational Tools:** To address situations where medications are not available at the bedside and enhance education efficiency, it was suggested to use medication photos or demonstration devices (e.g., inhalers) as teaching aids. | - **Development of patient education library:** - An electronic library of patient education materials was co-designed with the CP team, featuring medication leaflets with images or instructional videos. The materials were shared among CPs via the MS Teams portal and will be provided to patients upon ED discharge as printed copies or electronically via WhatsApp or email, based on patient preference. - A representative leaflet sample was reviewed by the PFAC chairman, who found it feasible and practical. - The library was based on a list of the most common ED discharge medications, identified via pharmacy dispensing system. - **Documentation and tracking:** Agreement with the CP team to document discharge education using current Cerner-based “Pharmacy Medication Education“ form. | - Patient education library |
| **Supporting Quotes** | *“What's the possibility for providing discharge medication on the bedside, for example the GCC (nationals), we can bring them the medication since there is no payment*” (N 8)  *“Maybe if it's next to the pharmacy (dedicated space for education), it will be easier for them. But if they have to go to another place, it will not be convenient”* (CP 3) | *“Another option is to give the education without having the real medications, like using samples or photos or empty boxes” (Pharmacy Lead)*  *“Culture wise, as Qataris, they don’t want to move, they won’t go there (clinic), they will go with bedside option”* (Pt. 1) |  |  |
| **Planning Post-Discharge Follow-Up and Handover Communication** | | | | |
|  | **Phase I Suggestions** | **Phase II (Co-design Workshop) Refinements** | **Post-Workshop Operational Integration** | **Final Output** |
| **Workflow** | - **Early patient engagement and expectation setting:**   ED CPs offer patients post-discharge follow-up visit in medication therapy management (MTM) clinic before leaving ED, confirm acceptance, and obtain preferred visit time and modality (face-to-face or virtual)   - **ED to MTM referral process:**   ED CPs refer patients to MTM clinic through Cerner.   - **Handover communication:**   ED CPs document a “handover” note in Cerner for ambulatory care CPs, highlighting key MTPs and follow-up priorities. | - The proposal was endorsed by all participants. | - **Preparation and feedback:** - The research team gathered input via email from participating pharmacists to identify preferred and feasible formats for inter-pharmacist handover communication. - Three proposed handover tools were submitted, the ISBAR tool (the hospital’s official tool [4]), The I-HAPPY checklist tool [5], and the American Pharmacists Association Transition of Care communication tool [6] - During CPs meeting, they emphasized the need to adapt existing tools to the local context, noting that none accounted for pending laboratory or microbiology results requiring follow-up in the MTM clinic. - **Documentation and tracking:**   A standardized handover note template was co-designed in a follow-up meeting and piloted in practice to assess feasibility. | - A Cerner-based handover communication note template co-designed and finalized by CPs **(Appendix S3).** |
| **Supporting Quotes** | *“Explain to the patients before they leave that we want to follow up with you. Find when the right time is to call, to be sure that you're not disturbing them”* (Pt. 1)  *“Let's say I'm discharging my patient from ED to the clinic; I will be highlighting the barriers that I found, things that should be followed-up, doses that have been recently changed, compliance issue with certain medication, cost issue”* (CP 5) |  |  |  |
| **Post-Discharge Follow-Up with Pharmacist-Led Medication Therapy Management (MTM) Clinic** | | | | |
|  | **Phase I Suggestions** | **Phase II (Co-design Workshop) Refinements** | **Post-Workshop Operational Integration** | **Final Output** |
| **Workflow** | - **Follow-Up Format and Frequency:**   Two follow-up visits scheduled within one month of ED discharge, conducted by phone as the default to enhance convenience and acceptance, unless a face-to-face visit was preferred or clinically required. CPs schedule additional visits as needed.   - **Escalation and Communication Pathways:** - If MTPs are identified, CPs may contact the discharging ED physician, who is most familiar with the case, or the lead ED consultant if unavailable. - Refer patients to Primary Health Care Corporation (PHCC) general practitioners for medication or laboratory follow-ups, in line with current practice. - Refer to specialty clinics if a patient has an established hospital care provider. - In urgent cases, refer to medicine rapid access clinic. - Uncertainty around escalation of care for clinically unstable patients encountered in the clinic. | - **Follow-Up Format and Frequency:**   Two follow-up visits scheduled within one month of ED discharge, conducted by phone as the default to enhance convenience and acceptance, unless a face-to-face visit was preferred or clinically required. Limit total visits to three/month to improve efficiency.   - **Escalation and Communication Pathway:** - If MTPs are identified, contacting ED physicians was rejected as impractical and beyond their scope, given workload and role limitations. - If urgent follow-up is needed, ED CPs arrange post-discharge follow-up visit with medicine rapid access clinic before discharge. - Maintain PHCC follow-up according to current practice. - Refer to specialty clinics for patients having established hospital care providers. - Refer clinically unstable patients or those with critical results to ED, through Cerner, after discussion with lead ED consultant. | - Agreement with the CP team to adapt Boston University’s Project RED phone call script to ensure consistency [7]. It was translated to Arabic by a CP and validated by two others. - **Documentation and tracking:**   Agreement with CP team to use the current Comprehensive Medication Management (CMM) note template for Cerner documentation following each visit. | - Arabic version of phone call script |
| **Supporting Quotes** | *”The doctor who prescribed the medication is the best one to be contacted, he can make a decision”* (ED Physician 6)  *“We empower the patients, tell them about the expectation from the physician they are visiting. Then, we see the patients again within one or two weeks and find the intervention done, very practical, very effective"* (CP 2) | *“If I'm an ED physician, and the clinical pharmacist contacted me and he said that patient X, you've seen him two weeks ago and medication has to be adjusted, and I'm seeing 80 patients per day. Do you think that it will be my responsibility to adjust the dose?”* (Hospital Executive) |  |  |

**Table S6. Mapping implementation barriers to implementation support strategies**

| **Implementation challenge** | **Relevant Domain(s)** | | **SELECT Intervention Functions^(TDF Domains)^** | **Identified Facilitators** | | | **Level** | **Target Audience** | **Final Operationalized Implementation strategies** **supporting overall program adoption and integration** |
| --- | --- | --- | --- | --- | --- | --- | --- | --- | --- |
|  |  |  |  | **Theory and evidence-informed implementation strategies** | | **Stakeholder-Informed Solutions** |  |  |  |
|  | **TDF** | **CTF** |  | **SELECT Strategies**  Based on **TDF** Domains | **ERIC Strategies**  Based on **CTF** Domains | **Stakeholders Suggestions** |  |  |  |
| Limited stakeholder awareness of pharmacists’ ToC roles | 1) Knowledge 4) Belief about capabilities 6) Beliefs about consequences 12) Social Influence | - **Organizational Characteristics (inner settings):**   -Implementation climate | - Education**^(1,4,6)^** - Enablement   **^(1,4)^**   - Incentivization   **^(4,6)^**   - Persuasion   **^(4,6)^**   - Coercion**^(4,6)^** - Training**^(1)^** - Environmental restructuring   `` **^(12)^** | **Education:**  -Conduct educational meetings  -Distribute educational material  **Enablement:**  -Use of champions (e.g., PFAC members)  -Prepare patients/ consumers to be active participants  **Persuasion** -Identify and use local opinion leaders (PFAC) -Use mass media  -Conduct local consensus discussions | - Assess for readiness and identify barriers and facilitators - Build a coalition - Alter incentive/   allowance structures   - Identify and prepare champions - Conduct local consensus discussions - Capture and share local knowledge - Conduct local needs assessment - Create a learning collaborative - Involve executive boards - Facilitation - Promote network weaving - Recruit, designate and train for leadership - Inform local opinion leaders - Tailor strategies - Access new funding - Identify early adopters - Organize clinician implementation team meetings - Conduct educational meetings - Promote adaptability - Use advisory boards and workgroups - Obtain formal commitments - Fund and contract for clinical innovation - Change physical structure and equipment - Mandate change - Develop a formal implementation blueprint - Involve patients/consumers and family members - Conduct cyclical small tests of change - Stage implementation scale up - Provide ongoing consultation - Audit and provide feedback - Visit other sites - Use an implementation adviser - Increase demand - Model and simulate change - Develop and implement tools for quality monitoring - Create or change credentialing and/or licensure standards - Use other payment schemes - Place innovation on fee for service lists/formularies - Conduct ongoing training - Purposely reexamine the implementation - Revise professional roles - Develop resource sharing agreements - Centralize technical assistance - Conduct educational outreach visits - Make billing easier - Develop and organize quality monitoring systems - Use mass media - Provide local technical assistance - Obtain and use patients/consumers and family feedback - Change record system - Alter patient/consumer fees - Use train the trainer strategies - Develop academic partnerships - Create new clinical teams - Facilitate relay of clinical data to providers - Start a dissemination organization - Work with educational institutions - Distribute educational materials - Develop educational materials - Develop disincentives - Change liability laws - Change accreditation or membership requirements - Use data experts - Change service sites | - Targeted awareness campaigns targeting patients and healthcare professionals - Development and distribution of patient- educational materials (pamphlets. Brochures) - Bedside educational prompts - Utilize huddles to educate and update ED staff about the program workflow | Organization, Patient, Provider | Patients, ED staff | **Intervention Function: Education**   - **Staff Education:**   - Conduct educational meetings (e.g., daily huddle and morning report) to educate and update staff about the program workflow (CPs, ED staff) and set clear expectations about the program  - Distribute educational materials about the program among involved staff   - **Patient and Family Education:**   - Distribute educational leaflets/brochures about the program  - Use media to encourage patients to bring medications to healthcare visits  - Utilize bedside educational reminders, such as a note or a sticker asking patients, “Did you receive discharge education?”  - Use readily existing Hamad Medical Corporation (HMC) approved barcodes or videos to provide patient and family counseling and education (e.g., insulin, inhalers)  **Intervention Function: Training**   - Collaborate with senior academics from Qatar University (QU) on developing and revising the program design and delivery - Conduct orientation sessions for intervention implementers and collaborators (e.g., pharmacists, nurses) - Train CPs on motivational interviewing to improve their patient counseling and education skills - Involve QU college of pharmacy students in intervention delivery (e.g., patient interviews to obtain medication history), under the supervision of CPs - Enroll CPs in HMC offered communication skills workshops/training   **Intervention Function: Enablement**   - **Staff Enablement:**   - Identify and prepare champions in ED  - Create communication channels (e.g., WhatsApp groups/emails) among stakeholders and key leaders  - Provide standardization tools for CPs delivering the intervention to ensure consistency of intervention delivery (e.g., training manual/ standardized documentation notes, phone call scripts)  - Conduct weekly rounds to nursing stations to obtain feedback  - Provide ongoing consultations; conduct regular team meetings for updates  - Dedicate CPs to be physically available in the ED  - Establish a standardized ED discharge pathway that integrates CPs   - **Patients Enablement:**   - Involve the patient and family advisory council (PFAC) committee, present the study plan and co-design results to the committee  - Offer patients the option to provide feedback to Nesma’ak (patient helpline), review Nesma’ak reports related to the program  - Provide flexible follow-up appointments times and modality (e.g., face-to-face or over phone), considering patient preferences  **Intervention Function:**  **Persuasion**   - Conduct local consensus discussions, involve key leaders and stakeholders in developing the program workflow - Update hospital leaders about the program’s plan and progress   **Intervention Function: Incentivization**   - Offer free phone consultations for patients during post-discharge follow-up visits in the medication therapy management (MTM) clinic   **Intervention Function: Environmental Restructuring**   - Design electronic templates for CPs’ documentation through Cerner (e.g., Handover communication note) - Utilize WhatsApp reminders for staff (e.g., inclusion criteria/ reminders about discharge referral requirements) - Call patients before post-discharge follow-up visits in the medication therapy management (MTM) clinic as a reminder - Use barcodes to provide patient educational materials/leaflets - Use medication photos to provide education if the actual medication is not available - Arrange for bedside medication delivery if feasible |
| Limited acceptance and engagement with the pharmacist-led ToC program by ED staff and patients | 3) *Social/*   *Professional Role and Identity*   4) *Belief about capabilities* 6) *Beliefs about consequences* 15) *Cognitive & interpersonal skills* | - **Organizational Characteristics (inner settings):**  -Readiness for implementation (Staff commitment)  -Implementation climate (Relative priority)   -Team and network Characteristics | - Education   **^(3,4,6,15)^**   - Incentivization   **^(3,4,6)^**   - Persuasion   **^(3,4,6)^**   - Coercion**^(3,4,6)^** - Environmental restructuring**^(3)^** | **Education:**  -Conduct educational meetings  -Distribute educational  **Enablement:**  -Use of champions (e.g., PFAC members)  -Prepare patients/consumers to be active participants  -Use a learning collaborative/community of practice (CoP)  **Persuasion**  -Identify and use local opinion leaders (PFAC)  -Use mass media  -Conduct local consensus discussions  -Mandate change  -Audit and provide feedback      **Environmental restructuring**  -Reminders  -Revise professional roles  -Change record systems  -Change service sites  -Create new clinical teams |  | - Interprofessional collaboration to establish a standardized ED discharge pathway integrating structured physician-pharmacist referral mechanisms and defined communication and escalation pathways for medication therapy problems (MTPs) - Ensure physical presence of CPs in target ED areas - Mandate integration of CP-led interventions in the discharge workflow - Person-centered flexible service delivery models - Targeted CP capacity building - Emphasize early patient and family engagement mechanisms | Organization, Patient, Provider | Patients, ED staff, CPs |  |
| Perceived discharge delays due to pharmacist involvement in ToC | 4) Emotion 6) Belief about consequences 8) Intention | - **Organizational Characteristics (inner settings)**   -Implementation climate (Relative priority)  -Structural characteristics | - Incentivization**^(6,8,13)^** - Persuasion**^(6,8,13)^** - Coercion**^(6,8,13)^** - Education**^(6,8)^** - Enablement**^(13)^** - Environmental restructuring**^(13)^** | **Education:**  - Conduct educational meetings  -Distribute educational materials  **Enablement:**  -Use of champions  -Public funding and contracting  **Persuasion**  -Conduct local consensus discussions  -Mandate change  -Identify and use local opinion leaders  -Audit and provide feedback  **Environmental restructuring**  -Reminders  -Revise professional roles  -Change record systems  -Change service sites  -Create new clinical teams  **Incentivization**  -Alter incentive/allowance structures:  -Work to incentivize the adoption and implementation of the ideal practice. |  | - Interprofessional collaboration to establish a standardized ED discharge pathway (described above) - Leadership alignment through of program value and institutional priority-setting - Performance monitoring and feedback systems - Resource allocation (workforce and physical space) - Technology-enabled education support tools | Organization, Patient, Provider | Patients, ED staff, CPs |  |
| **Workforce and resource pressures**  (limited staffing + high volume and complexity + increased burden and workload on staff as a consequence) | 6) Belief about consequences | - **Organizational Characteristics (inner settings)**   -Structural characteristics  -Readiness for implementation  (Staff commitment), Staff time)  -Other resources | - Education**^(6)^** - Incentivization**^(6)^** - Persuasion**^(6)^** - Coercion**^(6)^** - Environmental restructuring**^(6)^** | **Persuasion**  -Identify and use local opinion leaders  -Audit and provide feedback  -Conduct local consensus discussions  **Enablement**  -Public funding and contracting  **Environmental restructuring**  -Create new clinical teams |  | - Leadership alignment through of program value and institutional priority-setting - Resource allocation (workforce and physical space) - Utilization of supervised pharmacy support personnel (students, and interns) to augment workforce capacity | System, Organization, Provider | System, Organization, Provider |  |
| **Competing Priorities** of ED staff | 3) Social/Professional Role and Identity 3. Belief about consequences | - **Organizational Characteristics (inner settings)**   -Implementation climate  (Relative priority)  -Readiness for implementation  (Staff commitment) | - Education**^(3,6)^** - Incentivization**^(3,6)^** - Persuasion**^(3,6)^** - Coercion**^(3,6)^** - Enablement**^(3)^** - Environmental Restructuring**^(3)^** | **Education:**  -Conduct educational meetings  -Distribute educational materials  **Enablement:**  -Use of champions  -Public funding and contracting  -Alter payments to health workers  **Persuasion**  -Conduct local consensus discussions  -Mandate change  -Identify and use local opinion leaders  -Audit and provide feedback  **Environmental restructuring**  -Reminders  -Revise professional roles  -Change record systems  -Change service sites  -Create new clinical teams |  | - Interprofessional collaboration to establish a standardized ED discharge pathway (described above) - Leadership alignment through of program value and institutional priority-setting - Resource allocation (workforce and physical space) - Utilization of supervised pharmacy support personnel (students, and interns) to augment workforce capacity | System, Organization, Provider | ED staff,  CPs |  |
| **Care coordination** and **interprofessional collaboration** challenges | 3) Social/ professional role 6) Belief about consequences | **Organizational Characteristics (inner settings)**  -Structural characteristics  -Team and network characteristics  (Team and network self-organization, Teams, networks, and communications) | - Education**^(3,6)^** - Incentivization**^(3,6)^** - Persuasion**^(3,6)^** - Coercion**^(3,6)^** | **Education**:  -Conduct educational meetings  -Distribute educational materials  **Enablement:**  -Use of champions  -Prepare patients/consumer-s to be active participants  **Persuasion**  -Conduct local consensus discussions  -Mandate change  -Identify and use local opinion leaders  -Audit and provide feedback  **Environmental restructuring**  -Reminders  -Revise professional roles  -Change record systems  -Change service sites  -Create new clinical teams  **Incentivization**  -Alter incentive/allowance structures:  -Work to incentivize the adoption and implementation of the ideal practice. |  | - Interprofessional collaboration to establish a standardized ED discharge pathway (described above) - Leadership alignment through of program value and institutional priority-setting - Workflow-integrated documentation and tracking systems - Person-centered flexible service delivery models - Systematic patient and family engagement mechanisms - Advocacy for CP transitional care role and anticipated program benefits | System, Organization,  Provider | ED staff,  CPs |  |
| **Patient-related challenges to medication reconciliation and education in the ED (**limited knowledge of home medications, clinical instability, caregiver unavailability) | 1) Knowledge 4) Belief about capabilities 10) Memory, attention and decision process 12) Social influences 13) Emotions | **Organizational Characteristics (inner settings):**  -Structural characteristics | - Education**^(1,4,10)^** - Incentivization**^(3,4,13)^** - Persuasion**^(3,4,13)^** - Coercion**^(4,13)^** - Enablement**^(1,10, 12,13)^** - Environmental Restructuring**^(12,13)^** - Training**^(10)^** | **Education**  -Distribute educational materials  **Enablement**  -Prepare patients/consumers to be active participants  **Persuasion**  -Use mass media  **Environmental restructuring**  -Change service sites  -Reminders |  | - Create a culture of bringing medications or medication lists to healthcare visits and encounters - Person-centered flexible service delivery models - Family and caregiver engagement in discharge planning - Targeted CP capacity building - Flexible medication access solutions (e.g., bedside medication delivery) | System, Organization,  Provider | ED staff,  CPs |  |
| **Limited pharmacists’ awareness about community resources,** sponsorship, and charity support that may enhance access to medications, particularly for patients with financial challenges. | 1) Knowledge 2) | **External Context (outer settings)**  -External networks | - Education**^(1)^** - Enablement**^(1)^** - Training**^(1)^** | **Education**:  -Conduct educational meetings  -Distribute educational materials  **Environmental restructuring**  -Reminders |  | - Targeted CP capacity building - Enhance communication about communication about community support-services | Provider | CPs |  |

**Key**: TDF domains (1. Knowledge, 2. Skills, 3.Social/Professional Role and Identity, 4.Beliefs about Capabilities, 5.Optimism, 6.Beliefs about Consequences, 7. Reinforcement, 8. Intentions, 9. Goals, 10. Memory, Attention, and Decision Processes, 11. Environmental Context and Resources, 12. Social Influences, 13. Emotion, 14. Behavioral Regulation, 15. Cognitive and Interpersonal Skills )

**Color key for ERIC Strategy endorsement percentages**: ≥100%, 50-99%, 20-49%

**Abbreviations**: TDF: Theoretical Domains Framework; CTF: Care Transitions Framework; ERIC: Expert Recommendations for Implementing Change; TOC: Transition of Care; ED: Emergency Department; PFAC: Patient and Family Advisory Council; MTM: Medication Therapy Management; AWH: Al Wakrah Hospital; PHCC: Primary Health Care Corporation; MTP: Medication Therapy Problem. CPs: Clinical Pharmacists

**Fig. S1 Key process evaluation indicators**

**References:**

1. Duncan E, O'Cathain A, Rousseau N, Croot L, Sworn K, Turner KM et al. Guidance for reporting intervention development studies in health research (GUIDED): an evidence-based consensus study. BMJ open. 2020;10(4):e033516.

2. Booth A, Hannes K, Harden A, Noyes J, Harris J, Tong A. COREQ (consolidated criteria for reporting qualitative studies). Guidelines for reporting health research: a user's manual. 2014:214-26.

3. Masnoon N, Shakib S, Kalisch-Ellett L, Caughey GE. What is polypharmacy? A systematic review of definitions. BMC Geriatr. 2017;17(1):230. <https://doi.org/10.1186/s12877-017-0621-2>.

4. Hamad Medical Corporation. Handover Communication policy/procedure (CL 6102). CL 6102 Handover Communication.pdf. . Accessed 05/10/2023.

5. Attfield E, Swankhuizen MP, Bruchet N, Slavik R, Gorman SK. Improving the Clinical Pharmacist Handover Process in the Intensive Care Unit with a Pharmacotherapy-Specific Tool: The I-HAPPY Study. Can J Hosp Pharm. 2018;71(2):111-8.

6. American Pharmacists Association. American Pharmacists Association. Transition of Care Handoff Tool. . Washington, DC: Unknown. Available from: no URL available. Accessed

7. Agency for Healthcare Research and Quality Rockville MD. Postdischarge Followup Phone Call Script (Patient Version). March 2013. Available from: <https://www.ahrq.gov/patient-safety/settings/hospital/red/toolkit/postdischarge-phone.html>. Accessed 02/10/2023
