# Supplementary material for: Codesigning a pharmacist-led transition of care program in the emergency department: a qualitative participatory study
Source: Int J Clin Pharm. 2026 Aug 14;48(6):2322–38. doi: 10.1007/s11096-026-02210-3 (PMC13731452; doi:10.1007/s11096-026-02210-3)
Supplement: Supplementary file 2 [file 11096_2026_2210_MOESM2_ESM.docx]

**Supplementary File 2**

**Title:** Codesigning a pharmacist-led transition of care program in the emergency department: A qualitative participatory study

**Journal:** International Journal of Clinical Pharmacy (IJCP)

**Authors:** Authors: Eman Al Hmoud(1,2), Raja Barazi(2), Binny Thomas(2), Almunzer Zakaria(2), Lubna Andraous(2), Rasha El Enany(2), Palli Valapila Abdulrouf, Sameer Pathan(2), Hamde Nazar(3), Mohamed Izham Mohamed Ibrahim(4), *Muhammad Abdul Hadi(4)

**Authors’ Affiliations:**

1.	QU Health Sector, Qatar University, Doha, Qatar

2.	Hamad Medical Corporation, Doha, Qatar

3.	National Institute for Health Research (NIHR) Newcastle Patient Safety Research Collaboration, Newcastle University, Newcastle-upon-Tyne, United Kingdom

4. College of Pharmacy, QU Health, Qatar University, Doha, Qatar

***Corresponding author**

Muhammad Abdul Hadi

Professor of Clinical Pharmacy and Practice

Graduate Coordinator: PhD in Health Sciences (Clinical and Population Health Research)

Department of Clinical Pharmacy and Practice

College of Pharmacy, QU Health,

Qatar University

Doha, P.O. Box 2713

Qatar

Tel: 00974 4403 5582

Email: [mabdulhadi@qu.edu.qa](mailto:mabdulhadi@qu.edu.qa)

**Interviewer guide** for clinical pharmacists

| **Opening:** | |
| --- | --- |
| Welcome: Interviewer will welcome the participant.  General introduction: The interviewer will introduce him/herself.  Example  “Thank you for accepting to take part in this interview. My name is ___, a (job title) and research investigator at Al-Wakra Hospital.    We are planning to develop a pharmacist-led transition of care program that aims at improving continuity of care for patients discharged from the ED.  The main purpose of this interview is to explore your experience and perspectives around challenges to medication management around the time of discharge from the emergency department (ED). We would also like to explore your views and expectations of introducing the pharmacist-led transition of care (ToC) program.    This interview will help us in developing the program and enhancing its acceptability by seeking input of service users. | |
| **Ground roles:** | |
| 1. Please talk freely; there is no right or wrong answers.  2. Try to reflect on your experiences with the program.  3. Whatever is mentioned within the meeting will be completely anonymous and will not be linked back to you.  4. We will audio record the discussion to ensure we capture all your insights accurately, and to help us revisit the information during write out, if necessary. We will not identify anyone by name in our report, you will remain anonymous. The recordings will be used solely for research purposes and will be deleted once written down.  5. You may refuse to answer any question or withdraw from the study at any time.  6. The duration of this interview is about 60-90 minutes. | |
| **Domain1:**  Experience and views on medication management upon care transitions in the ED and the role of clinical pharmacists in enhancing this process. | |
| Experience and views on medication management upon care transitions in the ED: | - **Can you describe your overall experience with and views about specific challenges that may disrupt continuity of care and lead to suboptimal medication management during this period?** - **How do you think the process and challenges of discharging patients from the ED compares to discharging patients from other hospital units?** |
| Experience and views on the role of clinical pharmacists in enhancing the transition of care process:  Knowledge  Skills  Confidence  Belief about capabilities  Social/professional role and identity | - To what extent do you believe that participating in ToC process **is a legitimate part of your role as a clinical pharmacist?** - **What skills do you have as a pharmacist** that help you optimize transitions of care for high risk patients - **Confidence in delivering care within the context of ToC, both generally and specifically in the ED:** - In general, in what situations do you feel confident participating in the ToC process? What factors enhance your confidence? - Can you provide examples of experiences or training that have increased your confidence in managing ToC?(e.g., post discharge F/U) - Are there any specific situations where you do not feel as confident in your role in the ToC process? What contributes to this lack of confidence? - **To what extent do you believe that a ToC program is aligned with HMC/AWH values and priorities?** |
| **Domain 2:**  Eligibility criteria for a future pharmacist-led ToC program targeting patients discharged from the ED. | |
| - If a pharmacist-led ToC program is implemented in your facility**, who do you believe would benefit from such program the most?** (in other words, what are the highest risk ED transitions/patients? - **Which patients would you exclude?** | |
| **Domain3:**  **Expectations regarding clinical pharmacists’ interventions that should be included in the program**  **Domain 4:**  **Potential barriers and facilitators to implementing and integrating the program into the healthcare system, along with practical solutions to address any anticipated challenges.** | |
| **Knowledge, Environmental context and resources, Beliefs about consequences Memory, attention**  **and decision processes**  **Social/professional role and identity Social Influences**  **External Context**  **Organizational Characteristics**  (structural characteristics, networks and communications,  culture, climate, and readiness) | **The following are evidence based clinical pharmacist-delivered transitional care interventions that were shown to improve medication safety at care transitions:**   1. Medication history and reconciliation at discharge and communication of medication changes 2. Review of discharge prescription 3. Patient education and self-management promotion at discharge 4. Discharge planning: timely coordination and communication of complete information to health care team, use of hand-offs, and scheduling post-discharge visits (with a pharmacist clinic only?) 5. Post discharge follow up: continue education post-discharge, monitor and manage symptoms after discharge, and follow-up with outpatient providers   **How would you rank the Acceptability, Practicability, Effectiveness, Affordability, Side-effects, Equity for each of these interventions from 1 to 5, 1 indicates least and 5 indicates highest score.**  **Ask participants to provide a justification to back up their selection.  Now let’s explore potential barriers and facilitators for each of these interventions and suggestions to overcome challenges.  *The following prompts can be used, as appropriate  **Barriers:**   - **Knowledge:** Health literacy, language barriers, lack of understanding of importance of having education at discharge, lack of knowledge about pharmacist’s role at ToC - **Environmental context and resources:**   Task/ process standardization  Team and work characteristics, Teams, networks, and communications  Staff commitment  Relative/ competing priority in a busy ED  Authority: Pharmacists/ other physicians perceived and actual degree of authority to make decisions and act autonomously (  HIT systems: communications, electronic health records (e.g., completeness of documentation)  Presence of Physical space and equipment dedicated to or impacted by the intervention  Staff time dedicated to implement the intervention  Patient self-management infrastructure: Training, counseling, and education available to patients prior to the intervention  within the hospital and ambulatory setting  Leadership engagement (Whose involvement in this program do you think will be necessary to have maximum impact)  Other resources: Grant allocation, research assistant/ trainees  Patient priorities: patients are in a hurry to leave the ED at discharge   - **Beliefs about consequences:** potential delay in discharge process. - **Memory, attention, and decision processes:** patients are in a hurry to leave the ED at discharge - **Social/professional role and identity:** nurses don’t take responsibility to communicate with pharmacists about discharge plans, physicians’ resistance to accept pharmacists’ recommendations, Pharmacists are unable to place medication or lab orders - **Social Influences:** negative perception of the value and meaning of other staff roles   **Facilitators:**   - **Environmental context and resources, Memory, attention, and decision processes, Social/professional role and identity, Reinforcement**   Leadership engagement (Whose involvement in this program do you think will  Presence of Physical space and equipment dedicated to or impacted by the intervention  Staff time dedicated to implement the intervention  Other resources: Grant allocation, research assistant/ trainees   - **Skills/ beliefs about capabilities:** training of pharmacists - For post-discharge visits: - Delivery format (face-to-face versus in-person): why? - Frequency of follow-up visits (one visit or two, time frame after discharge)   Are there any other interventions that you consider relevant to improving ToC from the ED that were not discussed?   - **Steps that a pharmacist delivering ToC interventions should take to resolve any identified medication-related issues ( e.g., unintentional medication discrepancy/ MTP) ? (for doctors)** - Prompts: - What would stop a pharmacist putting those practical steps/strategies into place?   What would make it easier to do so? |
| **Program specifications**  Skills/ belief about capabilities  **Social influences**  Leadership engagement  Staff commitment | - What training/ resources would be helpful for you to maximize your role in such a future pharmacist-led ToC program? - Whose involvement in this program do you think will be necessary to have maximum impact? |
| **Behavioural regulation, Memory, attention, and decision processes** | - Can you **take me through how you would decide** to take the necessary steps to deliver/ resolve the issues (e.g., unintentional medication discrepancy/ MTP) identified during the proposed program? |
| **Domain 5:** Outcomes measures for the program. | |
| **Beliefs about consequences, goals** | What do you think should be measured as an outcome of such a program?  i.e. how would you consider that the intervention had improved ToC? What are the most important outcomes? |
| **Goals/ motivation** | What benefits do you expect such a program will have?  What are the possible unintended consequences do you expect such a program will have? |
| Are there any additional comments or recommendations you would like to share regarding the program?  Thank you for taking part in this interview. | |

**Interviewer guide** for Health Care Professionals (non-pharmacist)

| **Introduction** | | | |
| --- | --- | --- | --- |
| Welcome: Interviewer will welcome the participant.  General introduction: The interviewer will introduce him/herself.  Example  “Thank you for accepting to take part in this interview. My name is ___, a (job title) and research investigator at Al-Wakra Hospital.  We are planning to develop a pharmacist-led transition of care program that aims at improving continuity of care for patients discharged from the ED.  The main purpose of this interview is to explore your experience and perspectives around challenges to medication management around the time of discharge from the emergency department (ED). We would also like to explore your views and expectations of introducing the pharmacist-led transition of care (ToC) program.  This interview will help us in developing the program and enhancing its acceptability by seeking input from service users. | | | |
| **Ground Roles** | | | |
| - Please talk freely; there are no right or wrong answers. - Try to reflect on your experiences with the program. - Whatever is mentioned within the meeting will be completely anonymous and will not be linked back to you. - We will audio record the discussion to ensure we capture all your insights accurately, and to help us revisit the information during write out, if necessary. We will not identify anyone by name in our report, you will remain anonymous. The recordings will be used solely for research purposes and will be deleted once written down. - You may refuse to answer any question or withdraw from the study at any time. - The duration of this interview is about 60-90 minutes. | | | |
| **Domain 1:**  Experiences and views about challenges to medication management during care transitions from ED and pharmacists’ role in optimizing this process | | | |
| Experience and views on medication management upon care transitions in the ED:  TDF domains:  Knowledge (patients/ CP/ HCP)  Beliefs about consequences Social/professional role and identity  Environmental context and resources  Social influences  Emotion | | **Can you describe your overall experience with and views about medication continuity of care as patients move between ED and home.**   - **Examples of challenges experienced by patients.** - **Causes of these challenges.**   **How do you think the process and challenges of discharging patients from the ED compares to discharging patients from other hospital units?**   - Urgency and pace of the ED influence ToC differently - Characteristics and needs of patients discharged from the ED differ from those discharged from other hospital units? | |
| Experience and views on the role of clinical pharmacists in enhancing the transition of care process:  TDF domains:  Knowledge  Skills  Confidence  Belief about capabilities  Social/professional role and identity | | **To what extent do you believe that a ToC program is aligned with HMC/AWH values and priorities ? National Health Strategy/ HMC’s** accreditation priorities (PCC/ JCI)?    **What do you consider the clinical pharmacists’ responsibilities to be within the multidisciplinary team in optimizing patient safety and bridging the gap of care during care transitions?**  **What do you think are the strengths pharmacists would bring in terms of supporting patient care around discharge from ED?**  **Can you think of any situations where you’ve seen pharmacists involved in patient care around the time of discharge? What was your impression?**  **Confidence in pharmacists’ ability to deliver care within the context of ToC in the ED?”**  Prompts:  Situations where you feel most confident in pharmacists’ participation in the ToC process? What enhances your confidence?  Situations where you would feel less confident in pharmacists playing a role in the ToC process? What factors contribute to this? | |
| **Domain 2:**  Eligibility criteria for a future pharmacist led-ToC program targeting patients discharged from the ED.  CTF: Population needs and resources | | | |
| - **If a pharmacist-led ToC program in ED is implemented in your facility, who do you believe would benefit from such program the most? (in other words, what are the highest risk ED transitions/patients?** - **Which patients would you exclude?** | | | |
| **Domain3:**  Expectations regarding clinical pharmacists’ interventions that should be included in the program  **Domain 4:**  Potential barriers and facilitators to implementing and integrating the program into the healthcare system, along with practical solutions to address any anticipated challenges. | | | |
| **Knowledge, Environmental context and resources, Beliefs about consequences Memory, attention**  **and decision processes**  **Social/professional role and identity Social Influences**  **External Context**  **Organizational Characteristics**  (structural characteristics, networks and communications,  culture, climate, and readiness) | | | - **The following are evidence based clinical pharmacist-delivered transitional care interventions that were shown to improve medication safety at care transitions:** - Medication history and reconciliation at discharge and communication of medication changes - Review of discharge prescription - Patient education and self-management promotion at discharge - Discharge planning: timely coordination and communication of complete information to health care team, use of hand-offs, and scheduling post-discharge visits (with a pharmacist clinic only?) - Post discharge follow up: continue education post-discharge, monitor and manage symptoms after discharge, and follow-up with outpatient providers   **How would you rank the Acceptability, Practicability, Effectiveness, Affordability, Side-effects, Equity for each of these interventions from 1 to 5, 1 indicates least and 5 indicates highest score.**  **Ask participants to provide a justification to back up their selection.  As we go through each intervention we explore facilitators and barriers.  **Barriers:**   - **Knowledge:** Health literacy, language barriers, lack of understanding of importance of having education at discharge, lack of knowledge about pharmacist’s role at ToC - **Environmental context and resources:**   Task/ process standardization  Team and work characteristics, Teams, networks, and communications  Staff commitment  Relative/ competing priority in a busy ED  Authority: Pharmacists/ other physicians perceived and actual degree of authority to make decisions and act autonomously (  HIT systems: communications, electronic health records (e.g., completeness of documentation)  Presence of Physical space and equipment dedicated to or impacted by the intervention  Staff time dedicated to implement the intervention  Patient self-management infrastructure: Training, counseling, and education available to patients prior to the intervention  within the hospital and ambulatory setting  Leadership engagement (Whose involvement in this program do you think will be necessary to have maximum impact)  Other resources: Grant allocation, research assistant/ trainees  Patient priorities: patients are in a hurry to leave the ED at discharge  **Beliefs about consequences:** potential delay in discharge process. |
| **Program specifications:** | | | |
| - **For post-discharge visits:**   Delivery format (face-to-face versus in-person): why?  Frequency of follow-up visits (one visit or two, time frame after discharge)  Prompts:   - Where, when and how will you deliver each of the selected intervention components?   (best time, bed-side versus dedicated space, face to face vs. teleconsultations, frequency of clinic visits)   - What would stop you putting those practical steps/strategies into place? - What would make it easier to do so? - How would a pharmacist react if there is no identified healthcare provider for this patient post discharge? | | | |
| **Domain 5:**  Outcomes measures for the program. | | | |
| **Beliefs about consequences, goals**  **Goals/ motivation** | What do you think should be measured as an outcome of such a program?  i.e. how would you consider that the intervention had improved ToC? What are the most important outcomes?  What benefits do you expect such a program will have? | | |
| Closing | | | |
| Are there any additional comments or recommendations you would like to share regarding the program?  Thank you for taking part in this interview. | | | |

**Interview guide** for Patients (or their representatives)

| **Opening:** | |
| --- | --- |
| Welcome: Interviewer will welcome the participant.  General introduction: The interviewer will introduce him/herself.  Example  “Thank you for accepting to take part in this interview. My name is ___, a (job title) and research investigator at Al-Wakra Hospital (AWH).    We are planning to develop a pharmacist-led transition of care program that aims at improving continuity of care for patients discharged from the emergency department (ED).  The main purpose of this interview is to explore your experience with handling medicines after discharge from the ED and any challenges that you may have encountered. We would also like to explore your views and expectations of introducing the pharmacist-led transition of care (ToC) program.    This interview will help us in developing the program and enhancing its acceptability by seeking input of service users. | |
| **Domain1:**  Experience and views on medication management upon care transitions in the ED and the role of clinical pharmacists in enhancing this process. | |
| Experience and views on medication management upon care transitions in the ED:  Knowledge, confidence  Environmental context and resources  Beliefs about capabilities  Beliefs about consequences  Behavioral regulation  Emotions | Thinking back to the last time you or your family member visited the ED:  Did anyone review your home medicines with you? How was that experience?  How comfortable were you asking questions about your medicines during your stay in the ED?  Where changes about your medicines communicated with you?    Can you describe your experience with getting education about medicines at the time of discharge from ED?  Tell me about who explained your instructions  How clear do you think were the instructions for the discharge medicines explained to you?  Did you feel that there were medicines that you think may have been helpful for you to discuss at the time of discharge or soon after discharge, for examples, medicines for chronic illnesses that were not evaluated during your ED visit?  In your opinion, how does the environment of the ED affect the time and attention given to explaining your medicines?  How do you think we can improve delivering education to patients during ED stay and at the time of discharge from the ED?  Tell me about any problems that you had filling the prescriptions or taking the medications at and after discharge? For example, did you have problems paying for your medications or getting to the pharmacy to fill your prescription?  After leaving the hospital:  How easy or hard was it to understand what your medicine were for and how to take them?  To what extent you felt confident handling you medicines and taking them as prescribed?  Were there any challenges that stopped you from taking your medicines as instructed (example, fear or concern about side effects)?  How frequent do you feel that you are unsure, or need more information or clarification about your medicines after discharge? (example, drug interactions, timing of taking medicines, experiencing any side effects)  What difficulties did you encounter in accessing follow-up appointments after discharge?  How satisfied are you with the follow-up after discharge from the ED? (timing, frequency of visits, convenience of schedules) |
| Experience and views on the role of clinical pharmacists in enhancing the transition of care process:  Knowledge  Beliefs About Capabilities  Memory, Attention, and Decision Processes (recall) | Can you recall a time when a pharmacist helped you with your medicines, either in the hospital or after discharge? What was that experience like?  What do you think a pharmacist can do to help manage medications when a patient is in the emergency department?  What about after leaving a hospital ?  What expertise or skills do you think that pharmacists have to help in optimizing your medicines around time of admission or discharge? |
| **Domain 2:**  Eligibility criteria for a future pharmacist led-ToC program targeting patients discharged from the ED. | |
| Belief about consequences | Who do you think would benefit the most from a pharmacist-led transition of care program in the emergency department?  **Prompts:**   - A medical condition that needs additional care after you leave the ED (e.g., a new diagnosis of diabetes or heart failure). - Starting certain medicines that require close follow-up and monitoring (e.g., blood thinners). - Ongoing health problems (e.g., heart failure, diabetes, chronic lung disease, cancer). - Financial challenges or lack of resources (e.g., living in poverty, no health insurance). - Difficulty getting medical care after discharge (e.g., no regular doctor, long waiting time to be seen by a specialist) |
| **Domain3:**  Expectations regarding clinical pharmacists’ interventions that should be included in the program  **Domain 4:**  Potential barriers and facilitators to implementing and integrating the program into the healthcare system, along with practical solutions to address any anticipated challenges. | |
| Confidence  Beliefs About Capabilities  Belief about consequences  Emotion  Goals  **Knowledge, Environmental context and resources, Beliefs about consequences Memory, attention**  **and decision processes**  **Social/professional role and identity Social Influences**  **External Context**  **Organizational Characteristics**  (structural characteristics, networks and communications,  culture, climate, and readiness) | The following can be used as prompts:  Potential barriers:  Environmental context and resources: busy ED environment  Beliefs about consequences: potential delay in discharge process.  Knowledge: limited knowledge about home medicines  Memory, attention, and decision processes: patients are in a hurry to leave the ED at discharge  Social/ professional role and identity: belief that this is doctor’s responsibility  Leadership support  Potential facilitators:  Knowledge: Clear communication about the process  Environmental context and resources: timely availability of pharmacist, timely coordination and communication of complete information to health care team, dedicated space for education, utilize printable education material, video demonstrations, utilize emails/ smart phone applications  Memory, attention and decision process: Reminders, flexible appointments  **What kind of help from a pharmacist would be most useful for you when managing your medicines, either while in the emergency department or after you go home? (goals)**  **Prompts:**  **Medication history and reconciliation (at admission, discharge)**  How confident are you that a pharmacist could help you review your medicine and make a complete and accurate list of all your medicines when you visit ED or leave ED to home?  How do you think this process can help to improve the care provided to you in the ED?  (prompt: ensure there are no mistakes or missing information, avoid prescribing interacting drugs, develop better understanding about medical condition and medicines the patient is taking)  What do you think might make it difficult for a pharmacist to perform medicines review at admission or discharge from ED?  What can possibly make this process easier?  In your opinion, could these difficulties differ between the time of admission and discharge?  If you are requested to select one time point to receive such a service, which one would you prefer (admission/ discharge), why?  **Medication review (during hospital stay, at discharge)**  How confident would you feel about a pharmacist reviewing your medicines during stay in the ED and at the time of discharge?  What do you think could happen if a pharmacist is or isn’t involved in reviewing medicine during or after your hospital stay?  **Education at discharge from ED:**  How confident are you with pharmacists educating you about your medicines at the time of leaving ED?  How confident would you feel about taking your medications if a pharmacist explained everything to you before you left the hospital?  What do you think might make it difficult for a pharmacist to explain about your medicines to you before you leave the hospital?  In your opinion, what could make this process easier?  **Post-discharge follow-up:**  How would you feel about having a pharmacist follow up with you after you leave the hospital? Would that give you more peace of mind when taking your medicines?  What do you think a pharmacist can do to help with managing your medicines after you leave the hospital?  How confident are you that a pharmacist could help you manage your medicines after discharge? Why do you feel that way?  What do you think might make it difficult for a pharmacist to help patients manage their medicines after they leave the hospital?  In your opinion what would enhance the public acceptability of delivering this service?  Would you prefer face to face clinic visits or phone consultations?  What could be the best frequency of follow up visits? |
| **Domain 5:** Outcomes measures for the program. | |
| Beliefs about consequences, goals | What do you think should be measured as an outcome of such a program to determine its success?  Prompts:  Patient satisfaction  Reduction in readmissions |
| Are there any additional comments or recommendations you would like to share regarding the program?  Thank you for taking part in this interview. | |
